# A Modular Vision Language Navigation and Manipulation Framework for Long Horizon Compositional Tasks in Indoor Environment

**DOI:** 10.3389/frobt.2022.930486

**Published:** 2022-07-13

**Authors:** Homagni Saha, Fateme Fotouhi, Qisai Liu, Soumik Sarkar

**Affiliations:** ^1^ Department of Mechanical Engineering, Iowa State University, Ames, IA, United States; ^2^ Department of Computer Science, Iowa State University, Ames, IA, United States

**Keywords:** ALFRED, vision and language module, graph convolutional mapping, NLP language parser, depth and mask estimation, BERT, long horizon compositional tasks, robot navigation and manipulation

## Abstract

In this paper we propose a new framework—MoViLan (Modular Vision and Language) for execution of visually grounded natural language instructions for day to day indoor household tasks. While several data-driven, end-to-end learning frameworks have been proposed for targeted navigation tasks based on the vision and language modalities, performance on recent benchmark data sets revealed the gap in developing comprehensive techniques for long horizon, compositional tasks (involving manipulation and navigation) with diverse object categories, realistic instructions and visual scenarios with non reversible state changes. We propose a modular approach to deal with the combined navigation and object interaction problem without the need for strictly aligned vision and language training data (e.g., in the form of expert demonstrated trajectories). Such an approach is a significant departure from the traditional end-to-end techniques in this space and allows for a more tractable training process with separate vision and language data sets. Specifically, we propose a novel geometry-aware mapping technique for cluttered indoor environments, and a language understanding model generalized for household instruction following. We demonstrate a significant increase in success rates for long horizon, compositional tasks over recent works on the recently released benchmark data set -ALFRED.

## 1 Introduction

**FIGURE 1:**
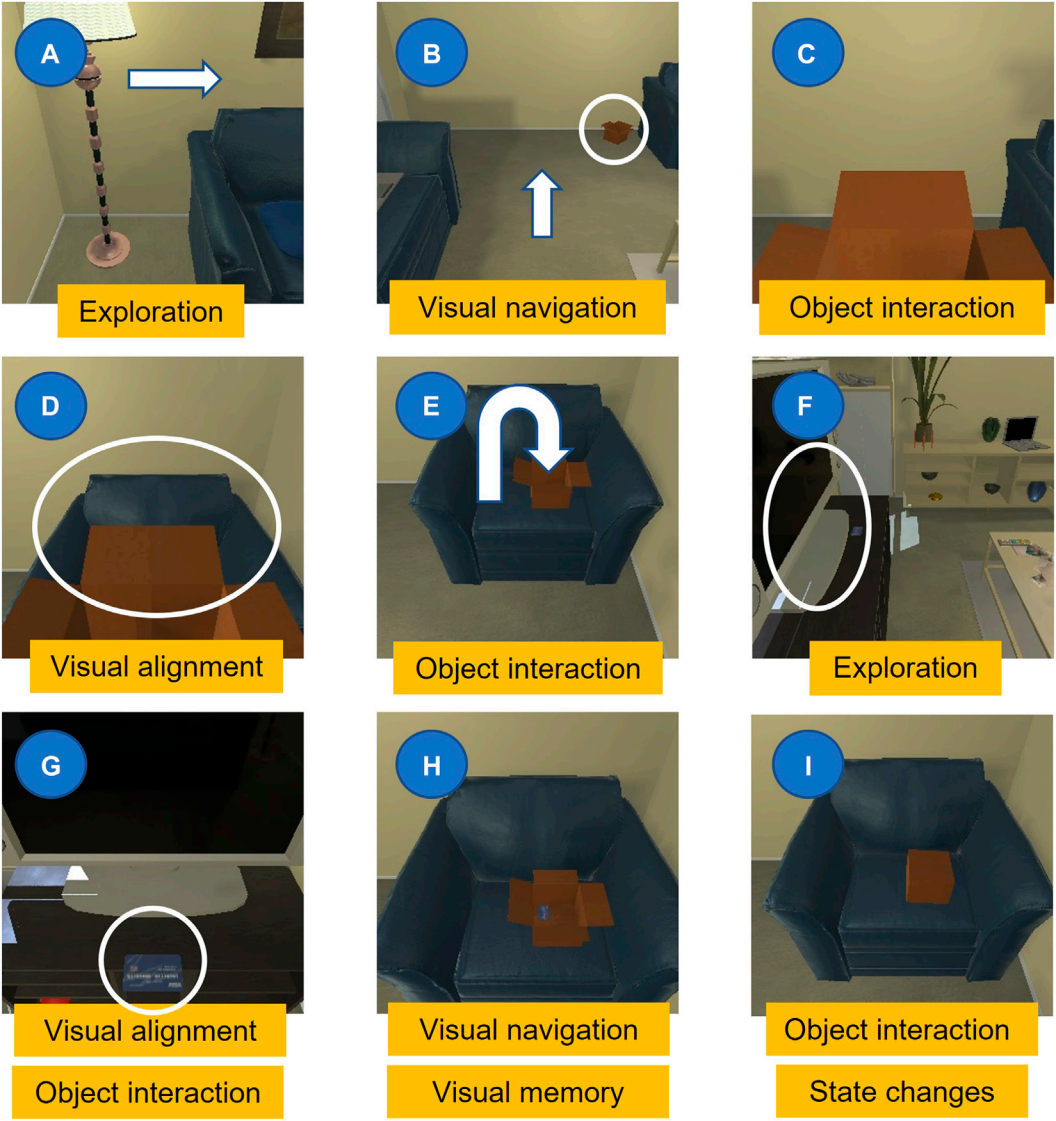
Example of a long horizon compositional task. An overall goal is provided to the robot through a sequence of instructions. Combining these with vision at the current time step, it must execute actions to satisfy the goal. Example: Goal: “Pick up a box, put it on a chair, put a credit card inside and close it.” **(A)**: “Turn towards the other side of the room with the blue chair in corner.” **(B)** “Walk across the room to the small box and pick it up.” **(C)** “Turn right and walk to the front of the blue chair in the corner.” **(D)** “Put the box down on the blue chair.” **(E)** “Turn around and walk to the front of the TV stand.” **(F)** “Pick up the credit card from the TV stand.“ **(G)** “Turn left and walk back to the blue chair in the corner, put the credit card in the box.” **(H)** “Close the box.“

Vision language navigation is interesting in its inherent Cross-Modal nature in anchoring natural language commands to visual perception, being highly relevant for practical robotic applications. Recent progress in deep learning for visual and linguistic representations pushed for ever more complex, close to real-life situations involving realistic simulators, longer execution trajectories, diverse object categories and natural language descriptions. Current techniques are being proposed for better visual navigation, improved language understanding, vision language grounding, and end-to-end differentiable planning. However, there remains a lack of holistic approaches to combine these elements in solving long horizon, compositional tasks with diverse object categories and irreversible state changes that require dynamic planning. Imagine having to follow a set of instructions in a visual environment as shown in Figure 1. While very intuitive for a human, generalization on such tasks in unseen environments has remained a hard problem.

Several past studies have tried to learn input language and action demonstrations through a joint action mapping using reinforcement/sequence-to-sequence learning techniques. However, judging from the nature of the language instructions used in these studies (e.g., usually using R2R (Anderson et al., 2018) data set), most often, agents are only required to identify a target location on the map and navigate to it. This situation can become much more complicated with compositional instructions common in household robotics, such as-“open the microwave, put the coffee, and wait for 5 s”. Although the number of expected actions from the language is not immense, the complexity of this joint mapping from vision and language to action may unnecessarily increase drastically. This in turn making existing reinforcement learning/sequence-to-sequence techniques much harder to learn.

A challenging benchmark data set, ALFRED, was proposed recently for vision language navigation and manipulation in household tasks (Shridhar et al., 2020). The initial study on this work achieved a very poor success rate, as low as 4%, even when deploying state-of-the-art learning frameworks (Supplementary Table S1 in Supplementary shows the difficulty level of this dataset compared to other datasets). Also, the recently published studies on the ALFRED dataset have not achieved a success rate of more than 30% on unseen environments. These works generally proposed various end-to-end deep learning approaches to train an agent to complete the defined tasks in instructions, which results in updating lots of model parameters in each training iteration. On the other hand, to predict the series of output actions more precisely, we proposed a modular-based deep learning framework in which each module focuses on executing specific intermediate information (e.g., the language module predicts the target object and navigation/manipulation commands from instructions which will be used in other modules). These modules are then connected to fulfill the whole task expressed in the instruction. In this paper, we make the following key contributions:

1) We propose a novel mapping scheme based on graph convolution networks to approximate the geometry of observed objects and navigable space around an agent in cluttered indoor environments, enabling improved navigation.
2) We propose a new application of state of the art natural language understanding models to better understand vision language instructions.
3) We extract depth and mask images of the robot’s environment panoramic image using adaptive depth estimation, and weighted semantic segmentation models.
4) Finally, we combine mapping and language understanding along with recent developments in semantic segmentation, monocular depth estimation and Cross-Modal grounding to arrive at a modular approach for state-of-the-art success rate on the newly released significantly challenging ALFRED dataset.

Here we present a review of all approaches used in vision language instruction following, divided into domains based on mapping, language understanding and end-to-end approaches, along with comparisons highlighting our novelty.

### 1.1 Mapping Based Approaches

Central to many robotic tasks is to learn and maintain a metric map to rely on for navigation. Early work by the authors (Smith and Cheeseman, 1986; Leonard and Durrant-Whyte, 1991), laid the foundations for the probabilistic formulation of building globally consistent maps, which is now widely known as Simultaneous Localization and Mapping (SLAM). Over potentially long explorations by the robot, data from a variety of sensors are integrated (Bongard, 2008; Fuentes-Pacheco et al., 2015). SLAM can be broadly divided into filter-based and graph-based. In the former, the temporal aspect of consecutive measurements is emphasized, whereas in the latter spatial aspects such as robot poses and visibility of landmarks are important (Grisetti et al., 2010).

In recent works for vision language navigation (Anderson et al., 2019), authors use a metric map as memory architecture for navigating agents. In topological maps, the graph convention can be used to represent environments with nodes representing free space with edges between the nodes connecting the free spaces by traversable paths. While metric maps focus on the geometry of the surrounding space of the agent, topological maps are more geared toward the connection between robot poses and trajectories in the environment, which often provides simpler solutions to route-based navigation (Werner et al., 2000). The introduction of semantics in graph-based maps can provide powerful solutions to encode relative locations of objects of interest in persistent memory (Pronobis and Jensfelt, 2012; Lang et al., 2014) and is useful when a higher level understanding of the surrounding is necessary. Deep learning approaches have also been proposed in this context, as in neural SLAM (Zhang et al., 2017) that tries to mimic the SLAM procedures into soft attention-based addressing of external memory architectures. Authors in (Chaplot et al., 2020) also extend upon these approaches in a modular and hierarchical fashion.

Several studies have also used differentiable and trainable planners for navigation (Tamar et al., 2016; Khan et al., 2017; Lee et al., 2018). There is another branch of work that focuses on accurately navigating to a location once a map has already been provided. They mainly focus on “targetted-navigation” and do not deal with the complexities of natural language. A major challenge they try to solve is to separate uncertain part from the certain part (e.g., for a given room layout where you already know the position of big items of furniture, where might small things like keys, remote control, etc exist) (Sünderhauf, 2019). This is an important concept when instructed language involves interaction with small objects in certain parts of an environment (not observable from far away, or may need to perform some actions to observe; such as open a drawer), not just navigating to a place. Although detailed topological maps may be provided, localization in dynamic settings may not be trivial. Authors in (Chen K. et al., 2019) explore a behavioral approach to navigation using a graph convolution network over a topological map. In this regard, our contribution is mainly constructing dense semantic topological maps from panorama images, specialized for cluttered indoor environments.

### 1.2 Approaches Based on Language Understanding

The earliest works on interpreting natural language commands for navigation have used statistical machine translation methods for mapping instructions to formal language (Matuszek et al., 2010; Chen and Mooney, 2011). In (Matuszek et al., 2010), the authors had used general purpose semantic parser learner (WASP) (Wong and Mooney, 2006) in order to learn semantic parser and constrain it with the physical limitations imposed by the environment. With the recent success of neural networks in natural language processing (Goodfellow et al., 2016), several techniques have been developed for interpreting user commands framed as a sequence prediction problem (Mei et al., 2016; Anderson et al., 2018). Such machine-level translation of natural language instructions has also been explored in the context of automatic question answering (Seo et al., 2016; Xiong et al., 2016). Following up, recently, authors in (Zang et al., 2018) use attention mechanisms to learn the alignment of language instructions with a given topological map and output high-level behaviors.

In contrast, we adopt a completely unique approach that associates semantics to each word of the language instruction using a semantic slot filling model. We find that the semantics we define are easy to learn and general enough to encompass various kinds of household instructions. This significantly reduces the complexity of input language instructions, allowing simple search techniques to execute these instructions on a semantic topological map (which we also learn using our new proposed technique-see mapping section). We will release the training data for this semantic slot filling model (that we use to fine-tune BERT). We believe that this will help researchers develop advanced techniques on top of our proposed instruction simplification mechanism.

### 1.3 End-to-End Learning Approaches

Several recent deep learning approaches propose to learn a mapping directly from inputs to actions, whether structured observations are provided (Mei et al., 2016; Suhr and Artzi, 2018) or the agent deals with raw visual observations (Misra et al., 2017; Xiong et al., 2018). Proponents of this approach argue about the simplicity of these models in outperforming a combination of models that require engineering hand-crafted representations. Cross-Modal grounding of language instructions to visual observations is often used in several works, via e.g., reinforcement learning (Wang et al., 2018, 2019), autoencoder architectures that impose a language instructions-based heat map on the visual observations (using U-net architectures (Misra et al., 2018), attention mechanisms (Zhu et al., 2020), or implementation of nonlinear differentiable filters (Anderson et al., 2019)). However, as we show later in the results, going end-to-end may not be best for generalizing to perform compositional tasks in unseen environments. Moreover, these frameworks often may not combine depth sensing and semantic segmentation, which are extremely valuable sources of information, and can be easily transferred from pre-trained frameworks. Also, keeping depth sensing and segmentation modules separate helps to incorporate knowledge about new unseen object categories into the framework in an easier fashion than in end-to-end models.

Recent attempts at using modular architectures for Alfred dataset include a research by Corona et al. (2020). In this study, the modular architecture consists of a high-level controller and eight independent modules for each subgoal task, which all have the same recurrent neural network architecture as Shridhar et al. (2020)’s monolithic model. Moca (modular object-centric approach) method (Singh et al., 2020) is also proposed on ALFRED dataset which two visual perception module (VPM) and action policy module are trained in an end-to-end manner. The VPM module in this study executes the interaction mask of the target object, and the APM module predicts the action sequence. HiTUT method (Zhang and Chai, 2021) tries to increase the success rate of the ALFRED dataset by decomposing task learning into three sub tasks; sub-goal planning, scene navigation and object manipulation. All three sub tasks share the similar input form; therefore they solve together by applying an unified model upon on multi-task learning. In HSLM (Blukis et al., 2022) paper, the proposed model architecture contains an observation model, high-level controller and low-level controller. The obsevation model constructs a semantic voxel map based on the RGB images of the environment which takes computation time. However, the step-by-step instructions in the dataset which are a useful source for accomplishing tasks are not used. Also, a hurdle in many end-to-end frameworks is combining all modules into end-to-end planning. Our modular consideration has a distinct advantage as it allows to incorporate several recovery strategies out of failure modes. Consider the connection from instructions (A-F) to instruction G in Figure 1. Having an independent mapping module communicating to an independent language and Cross-Modal understanding module allows to precisely relocate the blue chair in Figure 1 from instruction A in instruction G. In general, by quantitatively comparing these methods results with ours, we show that going end-to-end may not be best for generalizing to perform compositional tasks in unseen environments.

**FIGURE 2:**
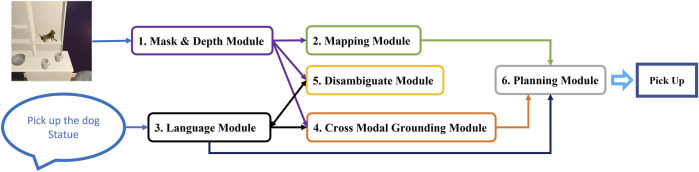
MoViLan overview: All six modules are shown in boxes. Arrows show how modules transfer information to achieve a set of actions (Pick up here).

In this work we proposed a new vision and language framework to navigate a robot to accomplish the household tasks of the challenging dataset—ALFRED. This framework contains six different modules; Mask and Depth module, Mapping module, Language module, Cross-Modal Grounding module, Disambiguate module, and Planning module as illustrated in Figure 2 (more detailed framework is represented in Supplementary Figure S1 in Supplementary). The functionality of each module, and how they build up the overall framework is explained in Section 2. Then the success rate of our framework for the whole task and for each subtasks besides comparison with related works are presented in Section 3. Finally, conclusion, limitations, and future works can be found in Section 4.

## 2 Materials and Methods

In Figure 2, we provide a summary of our modular approach to solve this problem that can be broken down into six major parts as:

### 2.1 Mask and Depth Module

As agent can navigate in the rooms virtually, it can capture a RGB image $x_{r}\in R^{h\times w\times 3}$ of its current observation. From the perspective of a robot, detecting objects and depths cannot happen without prior knowledge. Therefore, a need to give the robot perception to recognize the segmentation masks and depth of objects from a seen RGB image is called. This in turn helps robot to figure out which object it needs to interact and how far the objects are in the room. The first module in this work is presented to fulfill this need by using trained deep learning models which convert an RGB image *x*_*r*_ to corresponding depth map $x_{d}\in R^{h\times w\times 1}$ and segmentation mask $x_{s}\in R^{h\times w\times n}$ (each pixel stores a one hot encoding denoting one out of *n* possible object classes). Here, *h* is the height of the RGB image, *w* is the width, *n* is the number of object classes of interest.

**FIGURE 3:**
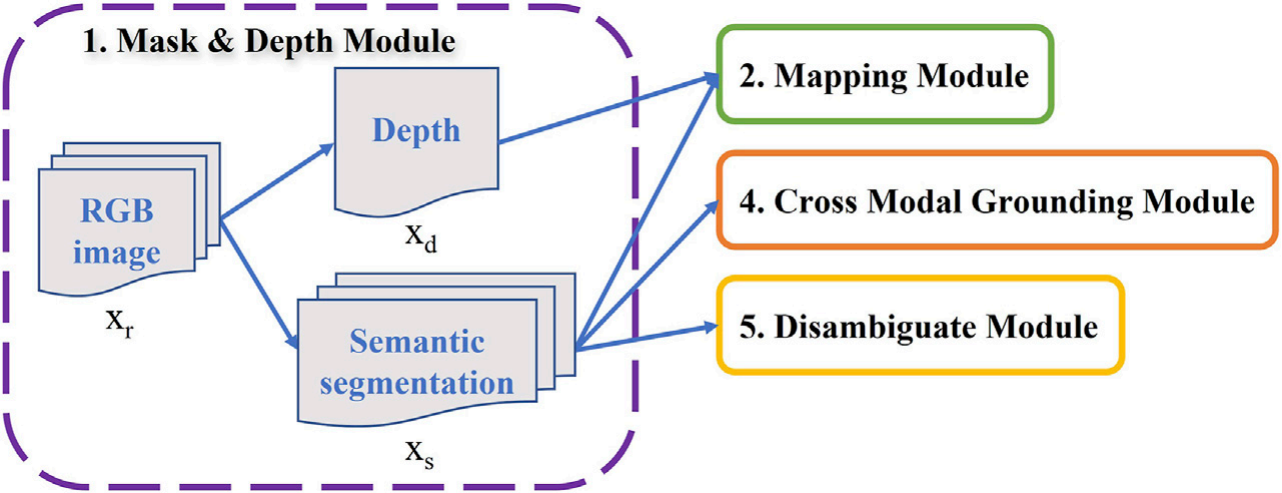
Mask and Depth Module: Predicts segmentation mask and depth map from robot’s current observation.

In the depth prediction used in this work, we borrowed Bhat et al. (2021) study on monocular depth prediction where the network adaptively concentrates on the region of the depth range which is more probable to occur. This depth prediction technique dividing the depth values into bins with flexible width. Then, the depth center values are adaptively estimated and depth values are calculated based on linear combinations of the bins center values. The output depth map *x*_*d*_ in our modular framework will be used in mapping module to generate navigation map as illustrated in Figure 3.

Semantic segmentation mask *x*_*s*_, another part of the Mask and Depth Module, is used as the input of three other modules (Disambiguate, Mapping, and Cross-Modal), indicating the importance of this model in our framework as it is shown in Figure 3. There are plenty of objects in all rooms of Ai2thor; however, we are looking for some special target objects which the robot interacts. Therefore, all 80 target objects from Alfred instructions were extracted and considered as corresponding classes for semantic segmentation. DeeplabV3 (Chen et al., 2017) is a semantic segmentation architecture that is used in this work because of its ability to handle the issue of segmenting objects at multiple scales. Some classes such as “Pot” or “Wine Bottle” are rare in the Alfred data, resulting in data imbalance problem. To address significant class imbalance in training data, we use a weighted loss function (Dice Loss). The weights are added based on the inverse numbers of objects in each class.

### 2.2 Mapping Module

**FIGURE 4:**
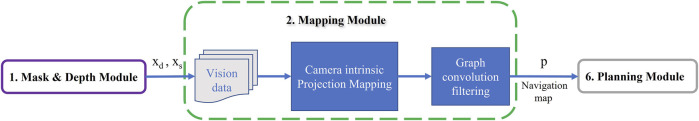
Mapping Module: Given depth map and segmentation mask, this module creates a projection (BEV) map and then it refines the map using the trained graph convectional network. The generated navigation map will be used further in the Planning Module.

Given an RGB image *x*_*r*_, a corresponding segmented image *x*_*s*_, and a corresponding depth image *x*_*d*_, this module first constructs an explicit Birds Eye View (BEV) map of the environment around the agent by approximate projection using geometric transforms and camera intrinsic parameters. Then, this map is refined to centimeter precise egocentric projection using our proposed filtering algorithm based on node classification using graph convolution. It considers the navigable space discretized in unit steps corresponding to the discrete step size taken by the agent at each time step. The steps of this module is represented in Figure 4.

#### 2.2.1 BEV Projection Map

Our goal is to obtain a projection $p\in R^{s\times s\times n}$ by considering having the segmentation mask and depth map of robot’s current observation image using the Mask and Depth Module. Here, *s* is the size of the spatial neighborhood around the agent where we are projecting to obtain a map. Each grid of the *s* × *s* neighborhood contains a 1 × *n* dimensional feature vector denoting one out of *n* possible object classes occupying the grid (including navigable space). Standard image segmentation and monocular depth estimation frameworks can be used to provide *x*_*s*_ and *x*_*d*_ from *x*_*r*_ with relative ease. Each pixel value of depth image can be multiplied with pixel indices, camera focal length and calibration matrix to obtain *x*, *y* and *z* distance of the pixel with respect to the agent, which is then rescaled and normalized to lie in any of the *s* × *s* grid around the agent.

Let $K\in R^{3\times 3}$ be the agent camera calibration matrix. Let *d*_*ij*_ denote the (*i*, *j*) pixel in the depth normal image *x*_*d*_ and *q* denotes camera focal length. Let *p*_*x*_(*i*, *j*) denote the *x* distance (relative to agent) of the (*i*,*j*)^*th*^ pixel in the RGB image, accordingly, *p*_*y*_(*i*, *j*) and *p*_*z*_(*i*, *j*). They can be obtained using the following formula.

$$\left[\begin{array}{c} p_{x}\left(i,j\right)\\ p_{y}\left(i,j\right)\\ p_{z}\left(i,j\right) \end{array}\right]=K\left[\begin{array}{c} i\\ j\\ q \end{array}\right]d_{ij},i\in \left\{1,\ldots ,h\right\},j\in \left\{1,\ldots ,w\right\}$$ (1)

Let *V* denote the maximum range of vision of the depth camera or depth estimation method, *D* denote the maximum pixel value of *x*_*d*_, and *r* denote the edge length of each small grid in the projection map (or resolution). A contributing element of the projection map $p\in R^{s\times s\times n}$ can be obtained from the pixel (*i*, *j*) as follows:

$$u\left(i,j\right)=\frac{V}{D\times r}p_{x}\left(i,j\right)-\frac{s+1}{2}$$ (2)

$$v\left(i,j\right)=\frac{V}{D\times r}p_{z}\left(i,j\right)-\frac{s+1}{2}$$ (3)

Therefore, pixel *i*, *j* in *x*_*r*_, *x*_*s*_ and *x*_*d*_ get assigned to the grid location *u*(*i*, *j*) and *v*(*i*, *j*) around the agent in $p\in R^{s\times s\times n}$ . Let (*u*, *v*) be the shorthand for each grid location which now stores a (1 × *n*) vector. Here *n* is the number of objects of interest. Now, let each element position *l* in the (1 × *n*) vector store 1 if the grid location was mapped from the contributing pixel in RGB image which belonged to class *n*, and 0 otherwise. Therefore, *p*_*uvl*_ can either store 1 or 0. Furthermore, we are using a panorama image for mapping, which means *p*_*uvl*_ is obtained for each small rotation of the agent *θ* that is taken to complete a full rotation 2*π*. Therefore *p*_*uvl*_ at a relative rotation of the agent becomes $p_{\mathrm{uvl}}^{\theta }$ . Let *R*(*θ*) be the transformation matrix that rotates a competing element by the angle *θ*. Then the final (1 × *n*) dimensional grid element of the projected map with grid index *u*(*i*, *j*), *v*(*i*, *j*) is given by:

$$p_{\mathrm{uvl}}=\frac{\theta }{2\pi }\underset{\theta }{\sum }R\left(\theta \right)p_{\mathrm{uvl}}^{\theta }$$ (4)

#### 2.2.2 Graph Convolution for Projection Refinement

**FIGURE 5:**
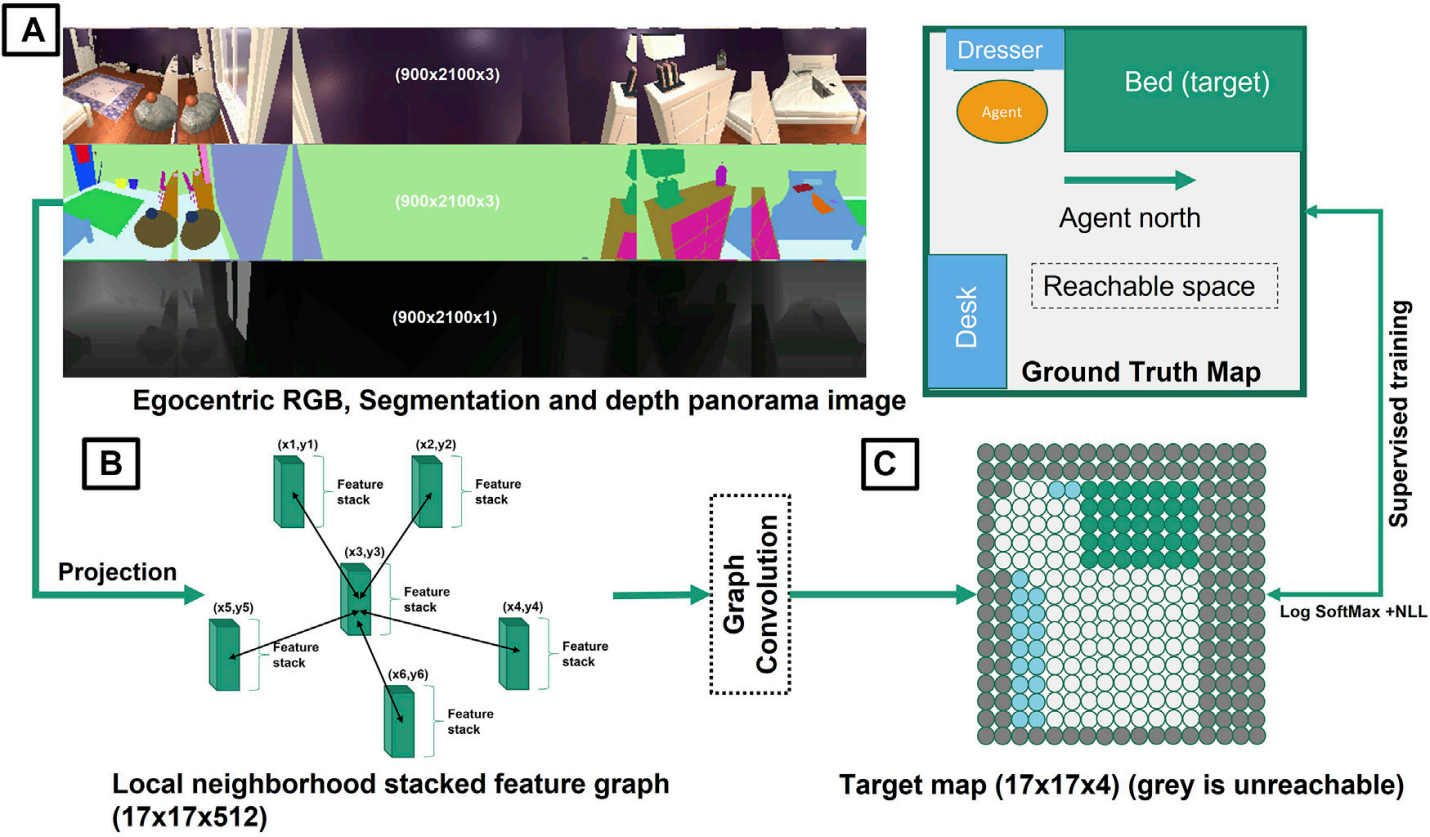
Schematic of mapping using graph convolution: **(A)** the agent obtains input panorama images in RGB, segmentation and depth modalities to construct a birds eye view (BEV) projection map of objects of interest around the agent using geometric transforms with camera intrinsic parameters (e.g., Bed (green), Dresser (light blue), and Desk (blue) and the navigable space (white) are the semantic objects of interest here). **(B)** Supervised training is used with the help of a true map (obtained by placing agent at each position in the room) for a select few rooms and agent locations. **(C)** the initial projection gets refined using a trained graph convolution network. Each grid node represents 0.25 × 0.25 m space.

The graph convolution algorithm we propose is essentially a filtering algorithm for refining obtained projection elements *p*_*uvl*_ in an (*s* × *s*) grid around the agent. (Figure 5).

##### 2.2.2.1 Preliminaries on Graph Convolution Networks (GCN)

GCN learns a function of signals or features on a graph G=(V,E) . The graph G takes as input:

1) A description of feature *x*_*i*_ for every node *i*, which can be summarized as an *N* × *D* feature matrix *X*, where *N* is the number of nodes and *D* is the number of input features.
2) A graph structure description in matrix form, supplied as an adjacency matrix *A*.

The graph G produces a node level output *Z* (an *N* × *F* feature matrix, where *F* is the number of output features per node). Let *H* ^(0)^ = *X*, *H* ^*l*^ = *Z*, and *L* be the number of layers of convolution, then the operation of neural network in the graph structure can be written as:

$$H^{\left(l+1\right)}=f^{l}\left(H^{\left(l\right)},A\right),l\in \left\{1,\ldots ,L\right\}$$ (5)

Implementation of different frameworks for graph convolution chiefly differs on the choice and parameterization of the function that needs to be learned *f* = {*f* ^1^, *…* , *f* ^*l*^}.

#### 2.2.3 Problem Specific Formulation

The graph structure we use is derived from the action space of the navigating agent in the form of a grid lattice (e.g., (Zhang et al., 2019)). Entire (*s* × *s*) space around the agent is converted to a grid topology with *N* = *s* ^2^ nodes with a connection between two nodes if the Manhattan distance between them is equal to 1. Two nodes are connected to each other irrespective of whether there exists a path between them or not. For our case, the path is a unit step that can be taken by the agent in any of the four directions. The input feature at each node *x*_*i*_ is equal to the (1 × *n*) dimensional grid element *p*_*uvl*_ discussed earlier, with number of input features *D* = *n*. The expected output at each node is a one hot encoding class wise representation for the following 4 possibilities (*F* = 4): **Unk** (e.g.,- beyond a visible wall)- encoded as [1, 0, 0, 0] **Nav**-encoded as [0, 1, 0, 0], **Target**-encoded as [0, 0, 1, 0] and **Obstacle**-encoded as [0, 0, 0, 1],

Applying graph convolution technique results in sharp and precise prediction of node (grid) coordination which is required for navigation. Moreover, by using GCN we can understand the relationship between nodes whether the path between them are navigable or not.

#### 2.2.4 Supervised Training

Algorithm 1 shows the details of forward computation and update of neural network parameters *f* for our proposed GCN. Let *L* be the total number of graph convolution layers, *f* ^*l*^ denote neural network parameters at a particular layer of GCN, *V* and *E* denote the collection of vertices and edges in the graph structure. First the node level features *x*_*i*_ are aggregated (summed together) over its connecting neighbors, after which a global function for that layer *f* ^*l*^ is applied to get global feature of the graph for that layer *l* as *Z* ^*l*^. The final global feature $\tilde{Z}$ is compared to the problem specific global feature labels (see paragraph above) using negative log likelihood loss function (NLLLoss), later all the parameters in all the layers *f* are updated using backpropagation with Stochastic Gradient Descent (SGD).

Algorithm 1 GCN computation + parameter update

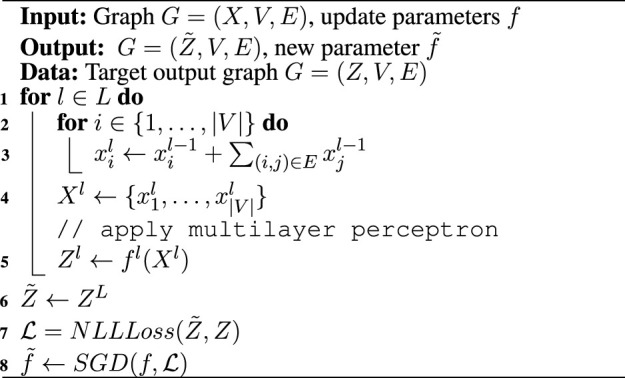

### 2.3 Language Module

This module takes the natural language instructions input to the agent. We propose generalized semantic labels for parsing sentences which can be trained using transfer learning from BERT with as few as 1,000 examples. This step helps the agent to decide targets for navigation, objects to interact with as well as relationship of the objects to each other with regards to fulfillment of the task (as understood from the language only).

#### 2.3.1 Contextual Understanding of Natural Language Instructions

**FIGURE 6:**
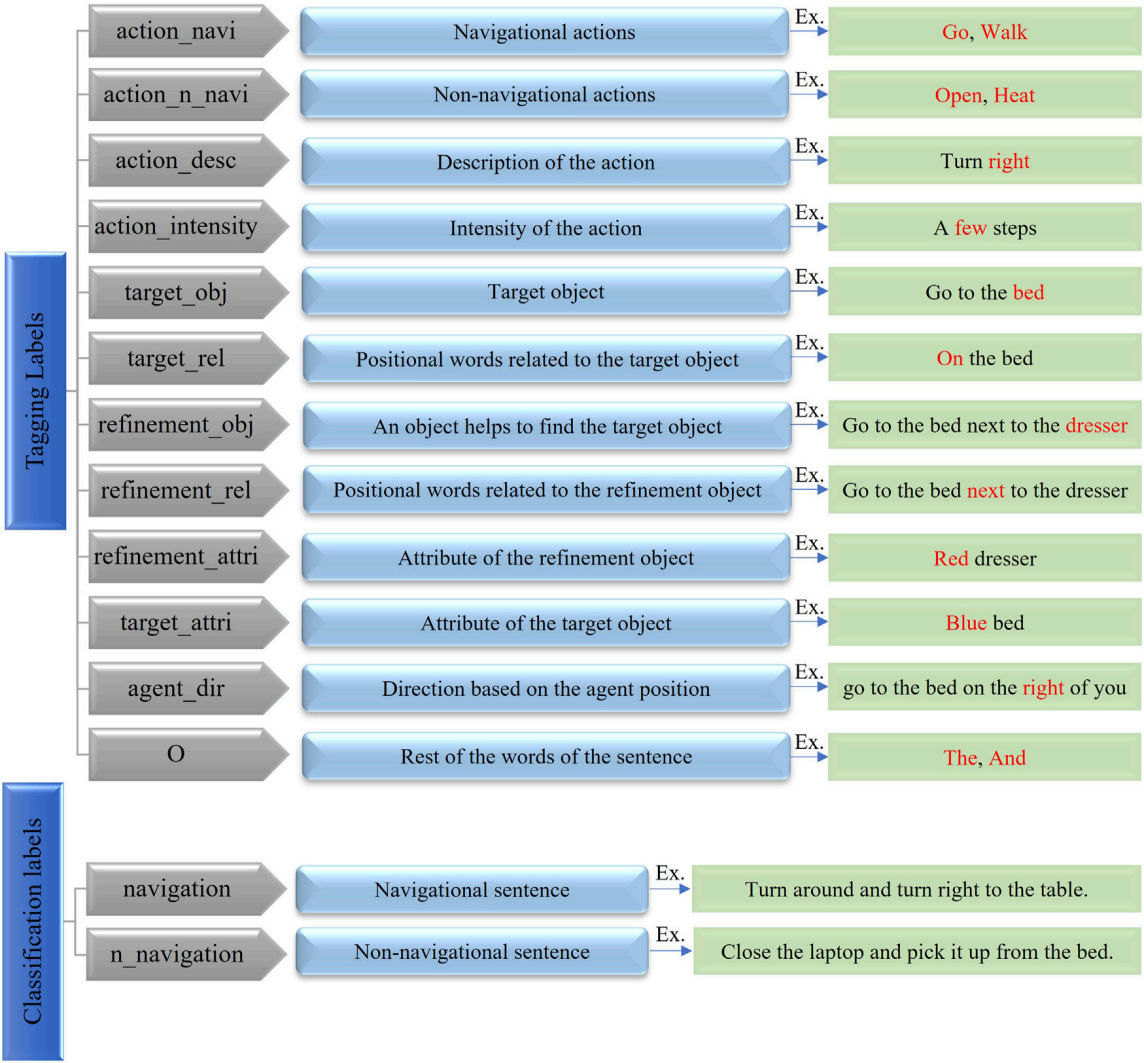
Defined labels for intent classification and slot filling tasks for ALFRED dataset.

We extract relevant interpretations of the input instructions that could be pieced together with information from the other modules. We focus on extracting two types of interpretations for the instructions simultaneously. First, each sentence is classified as either a navigation task or a non-navigation task denoted by *l* ^*i*^ (known as *intent classification*, with labels—navigation and non-navigation). Second, each word in these instructions is classified into a semantic category (slot labels), which indicate the role of a word and its semantic relations to the other words in the sentence (this is a sequence labeling task known as *slot filling*). Thus, the sequence of words in the input *x* = {*x*_1_, *…* , *x*_*T*_} is labeled with the slot label sequence $l^{s}=\left\{l_{1}^{s},\ldots ,l_{T}^{s}\right\}$ in the output. In the proposed language understanding module, two labels for intent classification and 12 labels for slot filling (word level semantics) are considered (see Figure 6). To simultaneously classify the intent and the label sequence of an instruction, we leverage the recently proposed Bidirectional Encoder Representations from Transformers (BERT) (Vaswani et al., 2017) approach. This model is used as a source model for transfer learning purposes (Chen Q. et al., 2019).

The input representation to BERT is a concatenation of WordPiece embeddings (Wu et al., 2016), positional, and segment embeddings. With a special classification embedding ([CLS]) inserted as the first token and a special token ([SEP]) as the final token, BERT takes the token sentence *x* = (*x*_1_, *…* , *x*_*T*_) as input and output hidden states *H* = (*h*_1_, *…* , *h*_*T*_). Based on hidden state of [CLS], the intent of a sentence is predicted as *l* ^*i*^ = softmax(*W* ^*i*^ *h*_1_ + *b* ^*i*^). The final hidden states of other tokens *h*_2_, *…* , *h*_*T*_ can be fed into a softmax layer to assign slot labels to words. Each tokenized input word is fed to the WordPiece tokenizer and the hidden state for the first sub token (call it *h*_*n*_ corresponding to *x*_*n*_) is input to a softmax classifier to get the label of the *n* ^*th*^ word. Let *N* be number of tokens, then:

$$l_{n}^{s}=softmax\left(W^{s}h_{n}+b^{s}\right),n\in 1,\ldots ,N$$ (6)

The following objective models the joint task of intent classification and slot filling

$$p\left(l^{i},l^{s}|x\right)=p\left(l^{i}|x\right)\overset{N}{\underset{n=1}{\prod }}p\left(y_{n}^{s}|x\right)$$ (7)

Finally, we maximize the conditional probability *p*(*l* ^*i*^, *l* ^*s*^|*x*), using Cross Entropy loss to train/fine-tune the model.

**FIGURE 7:**
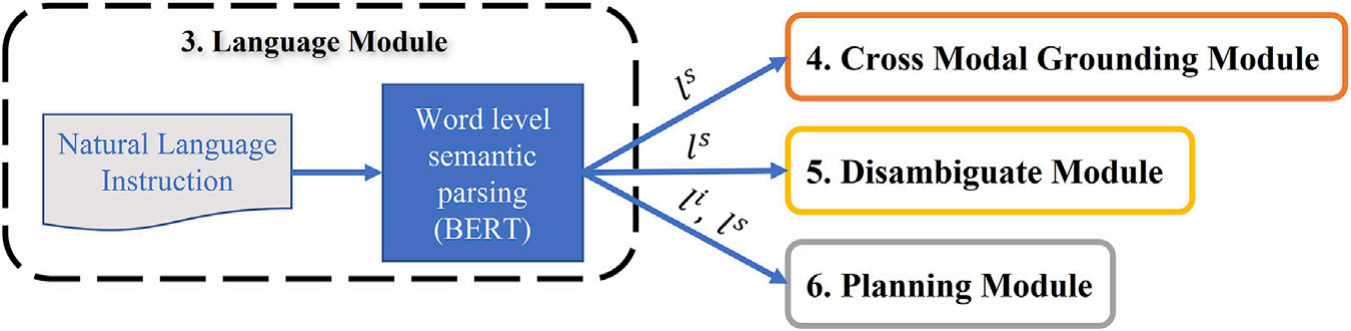
Language Module: The extracted intent and slot-filling from the fine-tuned Bert model is used in Cross-Modal grounding, Disambiguate and Planning modules.

Further, using the trained BERT-based language model, the intent *l* ^*i*^ and slot-filling *l* ^*s*^ of each language instruction are imported in Planning, Cross-Modal and Disambiguate modules (Figure 7).

### 2.4 Cross-Modal Grounding Module

This module identifies regions of the input RGB image that coincide with the natural language descriptions. This is achieved by combining image segmentation technique with word level semantics obtained from language understanding. Using such keywords from language to identify regions in image is known as referring expression image segmentation (e.g., (Ye et al., 2019)). We propose a simple restructuring of language inputs to LingUNet (Misra et al., 2018) and retrain it conditioned on semantic word labels provided by the language understanding module.

#### 2.4.1 Vision Language Grounding

A major challenge in vision language navigation is making the connection between language instructions and observed visual inputs. A technique that segments images based on key words in a natural language expression is known as referring expression image segmentation. We adapt the LingUNet learning framework (Misra et al., 2018) for predicting a probability distribution over input pixels of RGB image. We take a slight departure from the original formulation for presenting instruction encodings to a UNet architecture (Ronneberger et al., 2015).

**FIGURE 8:**
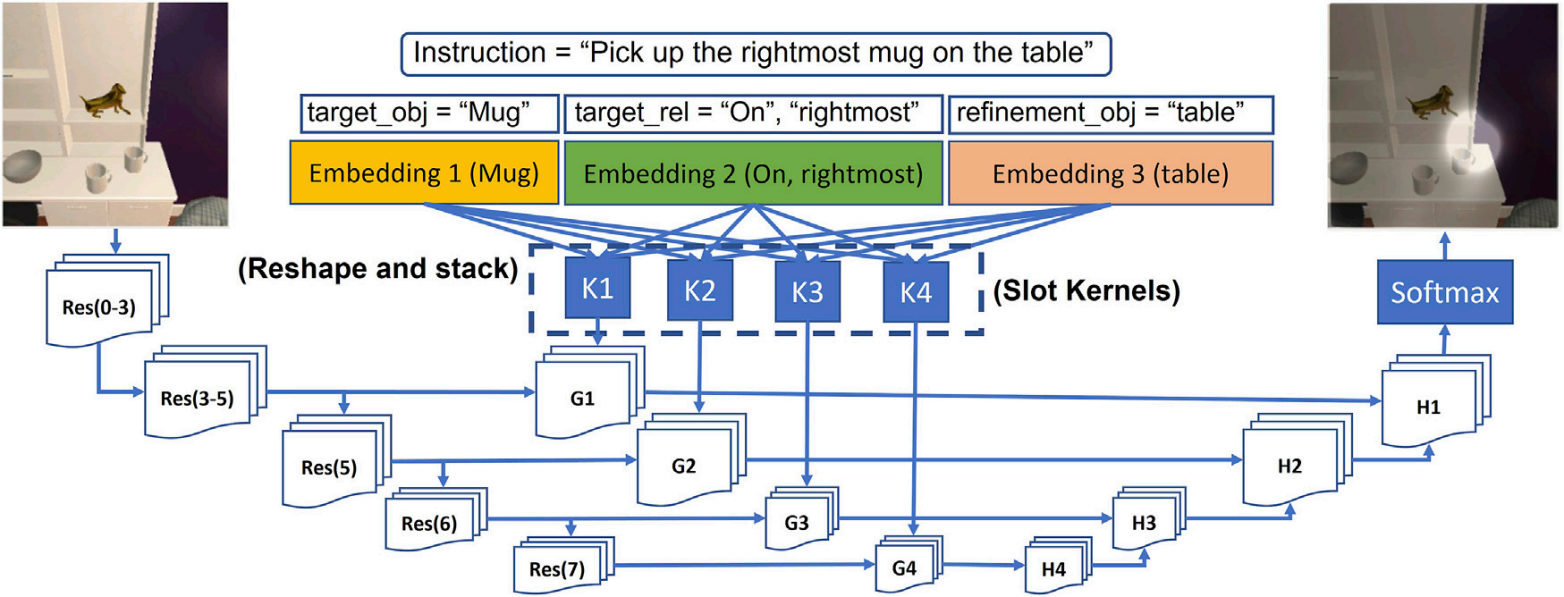
Cross-Modal grounding framework adapted from LingUNet. Res(i-j) denotes pretrained layers of ResNet starting from layer number i to layer number j. Output of softmax is intersected with output of UNet based class segmentation map to arrive at final map.

As shown in Figure 8, we pass the text instruction through the language understanding module which assigns slot labels to each word in the sentence according to the labels proposed in this paper (as shown in Figure 6). The words that get assigned labels are converted to embedding vectors through a learnable embedding layer. The embedding obtained for each slot label are reshaped into a kernel and stacked together as a set of filters (Ronneberger et al., 2015). Each of these stack of filters (*K*1, *K*2, *K*3, *K*4) are called slot kernels and are convolved over feature representations obtained from forward pass through the pretrained ResNet (He et al., 2016) layers. In the example shown in Figure 8, an image is extracted from the ALFRED dataset with Ai2Thor simulator. The input image gets a forward pass through the pretrained ResNet layers- *Res*(*i* − *j*), where *i*, and *j* are the layer numbers, and *i* − *j* implies the network formed by the layers *i* through *j*. Before upsampling through convolution filters *H*1 to *H*4, they are convolved with slot kernels to obtain *G*1 to *G*4. The final feature map, after application of Softmax operation, assigns a probability distribution which highlights regions/pixels *P*_*c*_ having a high probability of being the object mentioned in the text instruction.

**FIGURE 9:**
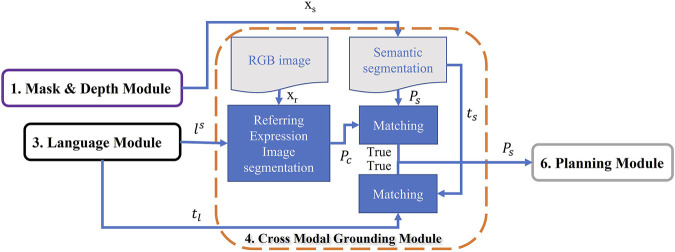
Cross-Modal Grounding Module: This module detects the pixels of object of interest extracted from language instruction using a vision language model (U-NET). The output of this module is further used for manipulation and navigation in Planning Module.

This prediction is further combined with segmentation mask to get the desired object segmented pixels. *t*_*s*_ is the object in the mask image *x*_*s*_ which its pixels in the mask image *P*_*s*_ mostly matches with the high probable pixels *P*_*c*_ extracted from UNet model. Moreover, *t*_*s*_ is then evaluated with the “target-object” *t*_*l*_ obtained from the Language Module. If *t*_*s*_ and *t*_*l*_ are not matched, the second most probable semantic mask is selected as *t*_*s*_. If no object is found, this instruction is reported as failure. The output of this module *P*_*s*_ is further used in Planning Module as it is shown in Figure 9.

### 2.5 Disambiguation

Image semantic segmentation is used alongside depth maps and RGB images in our algorithm. However, ambiguous natural language descriptions of a task can occur when an agent fails to recognize a different name for the same object visible in the scene. Ambiguous description can also occur when an object category which is completely new to a trained semantic segmentation model is expected to be related to a “target-object” mentioned in the language instruction. We propose a 2-way Disambiguation for these cases. This technique relies on the agents ability to connect to an online image database (eg-google image search) and download images corresponding to a specified keyword. Lets say for example, a language instruction specifies the agent- “Go to a dogbed”, but the image semantic segmentation model recognizes the object “beanbag”, and “beanbag” is visible to the agent. The 2-way Disambiguation can be briefly summarized below:

1) Disambiguate by pixel comparison
  a Download *N* images for the query object specified by the user in natural language (eg-“dogbed”)
  b Let there be *K* objects visible in the scene. For all objects visible in the scene, extract the group of pixels in the image corresponding to its object category as recognized the semantic segmentation framework.
  c Forward pass *N* images for query object through pretrained ResNet and obtain the feature vector as an embedding for the image. Call it *N*_*f*_ = {*N*_*f*1_, … *N*_*fN*_}. Forward pass *K* images for visible objects through pretrained ResNet to obtain *K*_*f*_ = {*K*_*f*1_, … *K*_*fK*_}
  d Calculate pairwise cosine similarity between each element of *N*_*f*_ and *K*_*f*_, and chose *K*_*fi*_ that has the highest value with all the elements of *N*_*f*_. The disambiguated object is the object corresponding to *K*_*fi*_.
2) Disambiguate by label comparison
  a Download *N* images for the query object specified by the user in natural language (eg-“dogbed”)
  b Let there be *K* objects visible in the scene. For all objects visible in the scene, query the name for the object category as recognized the semantic segmentation framework.
  c Download 1 image for each recognized object category with the query name as the segmentation class name (eg-“beanbag”), downloading a total of *K* images.
  d Forward pass *N* images for query object through pretrained ResNet and obtain the feature vector as an embedding for the image. Call it *N*_*f*_ = {*N*_*f*1_, … *N*_*fN*_}. Forward pass *K* images for visible objects through pretrained ResNet to obtain *K*_*f*_ = {*K*_*f*1_, … *K*_*fK*_}
  e Calculate pairwise cosine similarity between each element of *N*_*f*_ and *K*_*f*_, and chose *K*_*fi*_ that has the highest value with all the elements of *N*_*f*_. The disambiguated object is the object corresponding to *K*_*fi*_.

**FIGURE 10:**
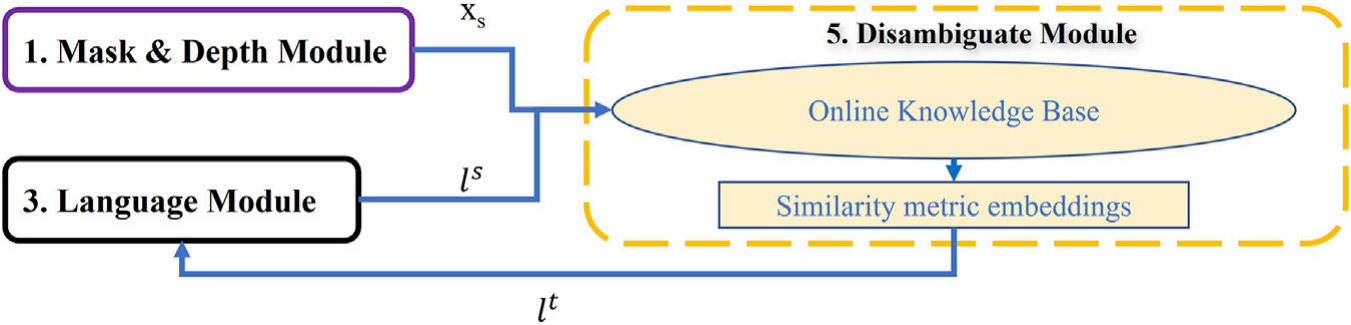
Disambiguate Module: This module searches for images of unclear object labels and try to assign recognized labels to the objects to reduce ambiguation.

Therefore, using this module results in extracting the consistent object name *l* ^*t*^ (which is similar to the segmentation labels) from the ALFRED complex instructions. This, in turn, is helpful to reduce confusion in interacting with correct objects in future steps. Figure 10 shows the schematic of this module.

### 2.6 Planning

**FIGURE 11:**
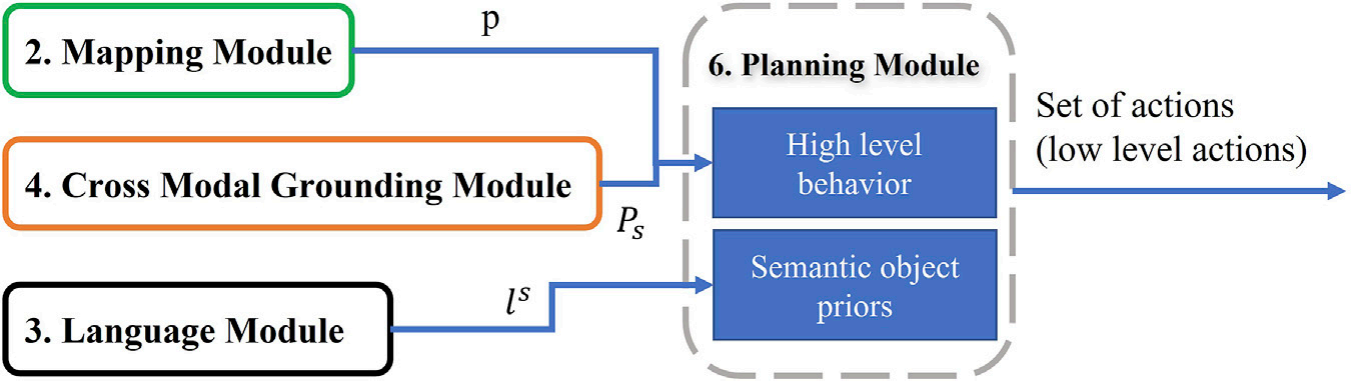
Planning Module: Given the navigation map, desired object pixels, and slot filling words from language instruction, this module can predict low level actions through two planning strategies: High level behavior and semantic object priors.

The goal of this step is to combine outputs from other modules and output a sequence of low level actions (Figure 11). First, it takes word level semantic parsing from the language module as input. After mining for associations across a large corpus of word slot labels this module assigns “semantic object priors” to each object identified in the language. This lets the agent understand for example, that small objects like “pen” are to be picked up using “Pickup” actions, whereas large objects like “table” might be suited for “Place” operation and so on. Output of semantic object prior along with the navigation map from the mapping module and matched pixels from Cross-Modal grounding module help the agent choose a high level behavior (such as “Pick up pen from table”). It is then converted to a sequence of low level actions using A* search algorithm with area (of the object of interest) maximization heuristic. Examples of low level actions would be- “MoveAhead”, “RotateRight”, “PickObject”, and “PutObject”, etc.

#### 2.6.1 Extracting Semantic Object Priors

The Language understanding module also provides a method to construct semantic relationships in between common household objects with regards to fulfillment of common household tasks which is used in the Planning Module. This kind of “common sense” relationships can be obtained in form of a densely connected knowledge graph (a small part of which is illustrated as an example in Supplementary Figure S8 in Supplementary). For example, this graph shows semantic relationships of the household object “Drawer” with several other household objects all of which are specified as nodes of the graph and edges describe the relationship between them. We propose a simple technique for automated extraction of these relationship patterns purely from language instruction data. Let $L=\left\{l_{1},l_{2},l_{3},\ldots ,l_{n}\right\}$ denote a corpus of language instruction data involving execution of tasks in indoor household environments. Let each *l*_*i*_ = {*s*_*i*1_, *s*_*i*2_, *…* , *s*_*i12*_} contain a list of slot labels (*s*_*ij*_) for each word in the instruction (Figure 6 for list of 12 slot labels). Our algorithm makes two passes over the corpus L . In the first pass, it identifies unique objects mentioned in the language by querying the slot label for “target-obj” and “refinement-obj” which are extracted by BERT. Each unique object is added as a node in the graph. For the same instruction, it also queries the slot labels “action-n-navi” and “action-desc” to determine the behavior of the agent expected towards the object such as whether it is generally something to be picked up (e.g., pen), or something which serves as a big landmark for the agent to navigate (e.g., bed). The words obtained by querying the slot labels for the instruction are added to a list of possible edge relationships which would be added later to connect the nodes of the graph. Same query can be made for the slot labels “refinement-rel” and “target-rel” to determine the location relationship (e.g., beside, above, below, and together) of the “target-obj” and the “refinement-obj”. The words obtained by querying the slot labels are again added into the list of possible edge relationships. In this way, after the first pass, the list of all nodes of the graph is obtained along with the possible edge relationships. However, these edge relationships can be highly repetitive, for e.g., the words “pick”, “grab”, and “take” describe the same relationship in the sentence “pick up the cellphone from the desk” for the node pair “cellphone” and “desk”. This is why we need to condense the edge relationships into as few distinct patterns as possible. For this, we use combination of several word embeddings and compare pairwise similarity. Although very rudimentary, this technique tends to work best for our scenario. A threshold value is determined based on repeated trials for a fixed corpus L and relationship pairs that have similarity values above the threshold are renamed into a common name (e.g., “pick” = “grab” = “take”). In the second pass of the algorithm, the nodes are then connected to each other through the refined relationships.

#### 2.6.2 Modular Planning for Execution of Low Level Actions From High Level Behaviors

In the final task for executing natural language instructions through low level actions, we propose a generic condition based planner. We proposed these conditions based on extensive study of 25,000 + natural language instructions and incorporated human like common sense reasoning through the conditions. Most of the components of the planer leverage specialized modules that can be trained independently using state of art deep learning models or customized using additional user specified domain knowledge. Because each of the tasks of visual perception, language understanding, as well as Cross-Modal Grounding and Disambiguation operate and can be trained independently, the bulk of planning boils down to choosing a high level agent behavior at a time step which is executed as a sequence of low level actions over several time steps. Here we provide a complete visualization of all the high level behaviors that are available to the agent in Supplementary Figure S9 in Supplementary.

The planner consists of two main modules—the navigation stack and the manipulation stack. Any instruction that has both navigation and manipulation components can be executed part by part as navigation and manipulation instructions. In the navigation stack, input navigation instruction can specify the agent to execute either a hard instruction such as “turn around”, “walk 2 steps forward”, arbitrary instructions, such as “walk forward a few steps” or targeted navigation such as “walk over to the desk”. These are high level behaviors which are exactly determined by querying slot labels corresponding to the parse of the instruction by the language understanding module. Hard and arbitrary instructions can be executed by direct application of low level commands as extracted by querying slot labels of the instruction.

On reaching targeted navigation, the agent utilizes the mapping module which relies on graph convolution filtering to provide an explicit BEV map of agent surroundings. However the number of targets *t* can be greater than 1, equal to 1, or even 0 when it is beyond depth perception or not visible to the agent, or is assigned a different object category (ambiguous instruction). When *t* > 1 the Cross-Modal Grounding module is invoked which matches groups of pixels in image to object descriptions in language to reduce the number of targets to 1. If *t* = 1, a straightforward A* planning can be invoked to enable the agent to reach the target location. If there is an obstruction not accounted for by the Mapping module (or moving objects), path planning may fail to execute, in that case, a small allowance is provided to the agent for a fixed number of collisions during which it remaps its surroundings. Popular techniques like Kalman filters can also be used to fuse different map readings over time. If the number of allowances is exceeded, the agent can try to face the target in a different direction (all objects have 4 different planning targets because of box like BEV approximation). Finally, when *t* = 0, the first check is done to ensure the object is visible in RGB frames (if its too far for depth perception). If visible in RGB, the agent makes a move in the general direction of the object considering the mapped navigable space around it. If the object is not RGB visible, a small neighborhood random exploration is done to make sure the object has not been cut off from visibility due to walls or other obstructions. If the object then becomes visible, Mapping module is called again to receive a new map. However, if the object is still not visible, the Disambiguation module (“Resolve”) is invoked, which relies on an internet connection to an online image search database to disambiguate objects and provide a valid target that matches language description. After navigation succeeds, a unit refinement step is executed in which the agent takes a unit step in the direction of the object which maximizes the segmented area of the object.

For the manipulation stack, the slot labels for the instruction is analyzed to decide whether the number of interaction objects *i* > = 2 (for example “Place the book on the table to the right of the lamp”) or *i* = 1 (for example “turn on the light”) or *i* = 0 (for example “gaze upwards”). In case *i* > = 2 an entity association is done based on slot labels which places objects in a hierarchy of interaction (for example table is level 0, lamp is level 1, book is level 2). After that, for both *i* > = 2 or *i* = 1, a check is done to make sure the objects mentioned in language are visible in RGB frame. If multiple objects of same category are present, Cross-Modal Grounding module is invoked, however if none of the objects are found to match with object categories for image segmentation, then Disambiguate module is invoked. Finally, after making sure the objects are visible, grounded and disambiguated, low level actions such as “pick (book, table)” or “turn-on(lamp)” can be executed.

## 3 Results

In this paper, we use the recently proposed ALFRED data set (Shridhar et al., 2020) built upon the Ai2Thor simulator (Kolve et al., 2017) to demonstrate the efficacy of our proposed MoViLan framework. Contrary to most other simulators, it features extremely long execution trajectories (some requiring upto 100 low level actions) and an immense object interaction diversity featuring compositional tasks such as in Figure 1, thereby allowing us to demonstrate performance in significantly harder and closer to real life scenarios.

Tasks Description—Scenarios are divided into collection of compositional tasks (language instructions with expert demonstrated sequence of low level actions -producing RGB images) over 120 different rooms including kitchens, living rooms, bedrooms and bathrooms (30 rooms each). Tasks demonstrated in ALFRED can be classified into 7 different types—Pick and Place, Stack and Place, Pick two and Place, Clean and Place, Heat and place, cool and place and examine in light. More closely, these tasks are a combination of 8 fundamental high level “sub-goals”- GoTo (navigate to some place), Pickup (pick up small objects like a pen), Put (place objects that have been picked up in a certain receptacle), Cool (cool an object), Heat (heat up an object), Clean (remove objects on a surface), Slice (cut an object) and Toggle (turn appliances on and off).

Training, Test, and Parameters—The available ALFRED dataset comes divided in train, test and validation folders over kitchens (1–30), living rooms (201–230), bedrooms (301–330), and bathrooms (401–430). We select language instruction data from the train folders to train our language module. Remaining modules (Cross-Modal grounding and mapping) do not require expert demonstrated actions to train upon and we simply collect RGB and depth data from each position of all the rooms for training our modules (rooms basic architecture remain unchanged across train and test, but language instructions and scene setup with placement of objects changes).

We compare performance with five other major baselines mentioned in baseline methods. While the baselines are tested on the tasks in the test folder, they are allowed to train on the remaining data set (train and validation folders). Across all the test folders, roughly 24,000 language instructions have been encountered.

Mask and Depth Predicition—RGB, mask and depth images extracted from training and testing data and different image augmentation techniques are used on training data for training this module. 8560 RGB images and their corresponding depth images are collected from 20 random locations in each room for training and testing. We provide further information regarding the training process and the outcome results in Supplementary (Supplementary Figures S2, S3).

Language Understanding—From our training data, we hand label 1,000 language instructions using our proposed labeling scheme and finetune on a pretrained BERT model for slot filing and intent detection tasks. Training parameters are the same as in (Monologg, 2020).

Mapping—On train rooms we scan panorama images at each navigable position in the room, approximate projection maps, and simultaneously form ground truth occupancy/collision maps (upon placement of an agent at each point in the room). The training of the graph convolution is to input approximate projections and estimate the corresponding collision grid map around the agent. (navigable space, obstacle, target, and unmappable (beyond wall)). Training parameters are same as in (Dgl, 2020).

Vision Language Grounding—BERT provides semantic slot labels for each word of the sentence. Therefore during test, instead of natural instructions, structured words can be provided as in Figure 8. Therefore to train LingUNet with our proposed input restructuring, human level natural language is not required. We can automatically generate training data as follows. Images (300 × 300 pixels) are extracted from each position of training rooms. From the ground truth segmentation of the image upto 3 objects can be selected randomly and their contours and center points are extracted. Their names are collected from the metadata of the simulation. Using a random ordering now each object can be referred to the other based on relative locations of their center points. Training parameters are same as in (Lil lab, 2020). No training is required for Disambiguation and Planning modules. To highlight our modules performance, qualitative results for Language, Mask and Depth, Mapping and Planning modules are mentioned in Section 3.2.

Agent Observation Space—Since Ai2Thor does not support panorama images during test time our agent acquires panorama images just as in the top left corner of Figure 5. Instance segmentation (middle) is estimated using UNet, and depth estimation (bottom) is used as provided by the simulator, which can also be replaced by off the shelf monocular depth estimation frameworks. The agent maintains a persistent map throughout execution of the provided instructions updated every time it takes an action using our proposed graph convolution filtering approach. On encountering collisions, it is registered into the persistent map and is recognized while taking subsequent actions.

Agent Action Space—From the simulator we chose a multitude of low level actions as listed in- (AI, 2020). Total of 13 discreet actions available each time step. For example, movement actions like—MoveAhead, RotateRight, LookUp, LookDown, and interaction actions like - Pickup, Put, Open, Close, ToggleOn, etc. Interaction actions require to either provide an interaction mask over input RGB image, or provide the complementary interacting object on which to act. While the *baseline* chose the former approach, we adopt the latter approach.

Metrics—We use the same metrics used for evaluating the *baseline* in (Shridhar et al., 2020): Task Success is a binary metric tracked for each task. Value is set to 1 if all the “sub-goals” that constitute execution of a complete task specified by the entire language instruction have been fulfilled. Otherwise this is set to 0. “Success-rate” of an algorithm is then calculated by taking the average of all task success values over all the tasks. Goal-Condition Success is the ratio of “sub-goals” completed successfully out of all the goals in the task. The sub-goal success of an algorithm is similarly an average over all tasks. Path weighted success rate and goal-condition success are computed as multiplying the task success and the goal-condition success rates respectively by the ratio $\frac{r}{R}$ , where *R* is the maximum of number of required actions (expert demonstrated) and number of low level actions executed by the agent, and *r* is the number of agent actions. Finally, Sub-Goal success rate is the ratio between the number of times a sub-goal is achieved and the total number of sub-goals.

### 3.1 Benchmark Comparison

Tables 1, 2 provide performance comparison for complete tasks and modular sub tasks respectively. In “MoViLan + PerfectMap”, ground truth BEV maps are provided to the agent as an ablation study for removal of our mapping module. Our framework demonstrates superior performance compared to the *baseline* (Shridhar et al., 2020) algorithms, *Moca* (Singh et al., 2020), *HiTUT* (Zhang and Chai, 2021), *HLSM* (Blukis et al., 2022), and *LWIT* (Nguyen et al., 2021) on complete tasks. For sub-goal tasks, our framework has significantly higher path weighted success rates for “GoTo” compared to previous works (language instructions requiring pure navigation) because of novel mapping module, and hence higher overall success rates due to better positioning. With perfect map, our other subgoals are comparable to SOTA, but have lower scores without in some cases (e.g. “Put”, “Pick up”) because of additional checks required by our planning algorithm to ensure better connection between individual sub tasks forming a major task (see Supplementary Figure S9). Note that with PerfectMap, task success rate increases significantly without any considerable increase in sub-goal success rate for “Slice”, and “Toggle”. This is because without good positioning, the agent would fail on many manipulation tasks.

**TABLE 1:** Task and Goal-Condition success percentages (rounded to nearest integer percentage). Results for the corresponding path weighted metrics are provided in parenthesis.

Test (unseen) (%)	Task		Goal-Cond	
SEQ2SEQ Shridhar et al. (2020)	0.5	(0.2)	7.1	(4.5)
SEQ2SEQ + PM Shridhar et al. (2020)	0.4	(0.1)	7.0	(4.3)
Moca Singh et al. (2020)	5.3	(2.7)	14.3	(10.0)
LWIT Nguyen et al. (2021)	8.4	(5.1)	19.1	(14.8)
HiTUT Zhang and Chai (2021)	13.8	(5.8)	20.31	(11.51)
HLSM Blukis et al. (2022)	20.3	(5.5)	30.3	(9.9)
MoViLan	27.1	(12.4)	35.2	(20.6)
MoViLan + PerfectMap	45.2	(21.8)	55.7	(28.3)

**TABLE 2:** Sub-Goal success rates (rounded to nearest integer percentage) on validation set.

Validation (unseen) (%)	GoTo	Pickup	Put	Cool	Heat	Clean	Slice	Toggle
SEQ2SEQ Shridhar et al. (2020)	21	20	51	94	88	21	14	54
SEQ2SEQ + PM Shridhar et al. (2020)	22	21	46	92	89	57	12	32
Moca Singh et al. (2020)	32	44	39	38	86	71	55	11
LWIT Nguyen et al. (2021)	39	79	66	85	94	95	68	66
HiTUT Zhang and Chai (2021)	—	71	69	100	97	91	78	58
HLSM Blukis et al. (2022)	—	57	—	39.6	17.0	25.8	—	—
MoViLan	45	50	50	66	68	50	57	60
MoViLan + PerMap	70	80	70	81	90	88	55	62

**FIGURE 12:**
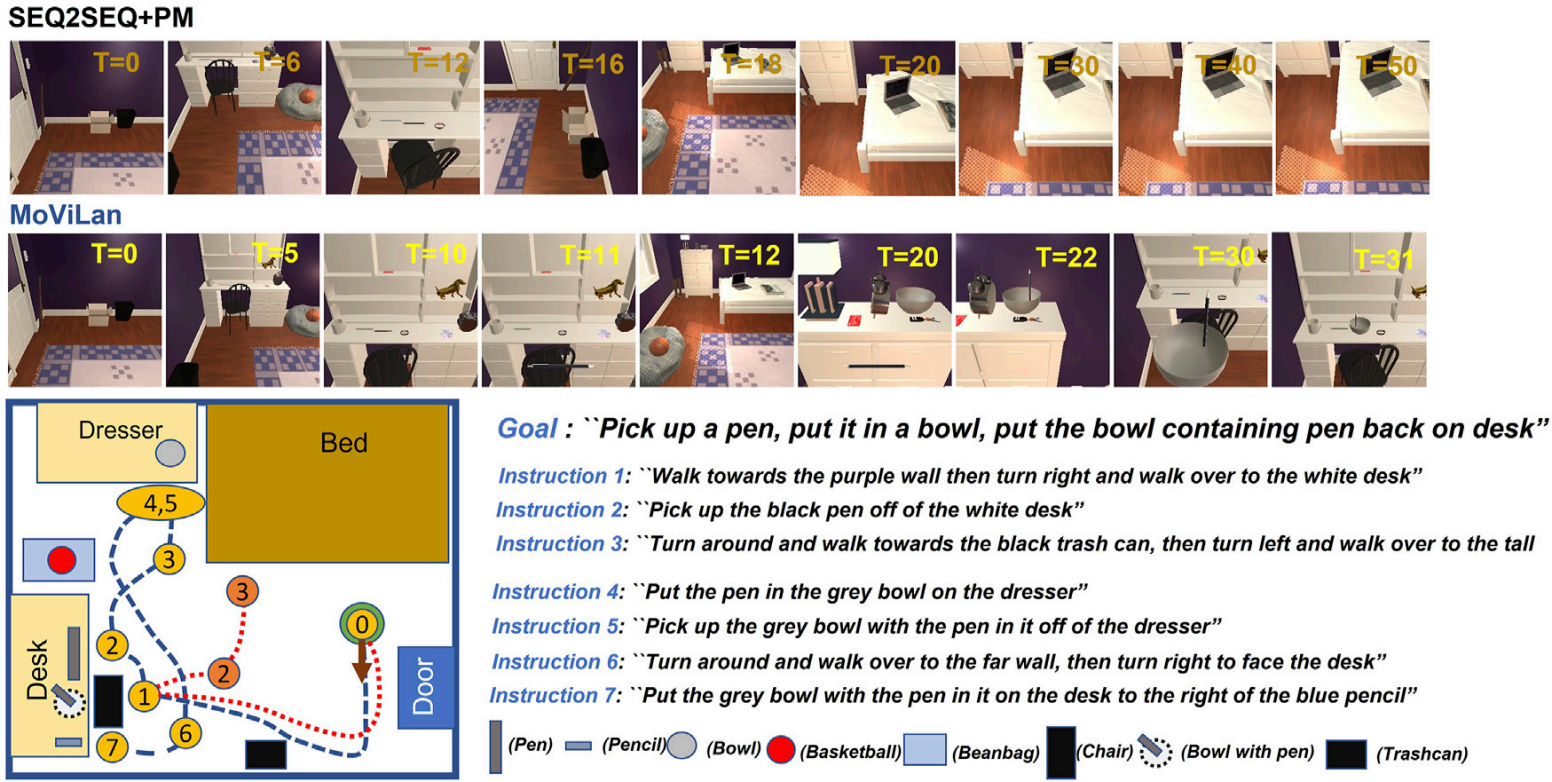
Comparison of trajectories for the baseline and our methods; agent shown by green circle, with starting orientation shown by brown arrow; relevant subgoals corresponding to instructions are shown in yellow (ours) and orange (author) colored circles; trajectory for our method shown by dotted blue line (success) and that for baseline shown by dotted red line (failure).

Anecdotally, Figure 12 demonstrates a failure case (top row) for *baseline* and a corresponding success case (bottom row) for our framework. It is observed that the *baseline* exhibits a behavior of “memorizing” trajectories from training data. While it leads to trajectory lengths close to expert demonstration in a small number of success cases, it also succumbs to failure in unseen rooms and for unseen object interactions. This conjecture is further supported by the fact that we observe *baseline* success rate as high as 25% on the training set. Authors in (Shridhar et al., 2020) have also highlighted the higher success rates for *baseline* on sub-goal tasks. This is an indicator that models using modularity or hierarchy would be favorable as we find in our case which is also obvious from our performance in Table 1 comparing to other methods.

### 3.2 Ablation Studies

Besides our framework’s overall success rate on the ALFRED dataset, here we report outcomes of some ablation studies to understand the roles of different modules. We perform these studies by replacing the modules with their corresponding ground truth, extracted from ALFRED. Small improvement using ground truth suggests strong performance by the corresponding module.

Ground truth depth map and segmentation mask are replaced in our framework and denoted as “+ gt depth” and “+ gt mask” in Table 3. Based on the results, the ground truth depth does not change the success rate significantly (3.2% on validation unseen) which means our predicted depth images are close to the ground truth. Given the high number of objects in the images and unbalanced class distribution of objects, the ground truth segmentation provides 14.3% improvement compared to our segmentation model. Also, the performances of depth and mask models are computed and shown in Supplementary Table S2 in Supplementary based on IOU metric for segmentation and RMS metric for depth.

**TABLE 3:** Ablation study on validation unseen dataset.

Validation (unseen) (%)	Task		Goal-Cond	
MoViLan + PerfectMap	43.5	(25.7)	56.1	(30.2)
+ gt depth	46.7	(23.7)	59.8	(31.1)
+ gt mask	57.8	(28.9)	68.1	(36.2)
+ gt mapping	49.0	(26.6)	61.3	(32.2)
+ gt language	50.6	(24.8)	64.2	(31.2)
+ gt cross-modal	53.3	(25.3)	68.5	(33.6)
− disambiguate	40.5	(24.6)	52.2	(28.5)

Comparing the performance of our framework with the case where the ground truth values for each module are replaced. Results for the corresponding path weighted metrics are provided in parenthesis.

Ground truth BEV view or projection map is extracted from the ALFRED dataset by considering the actual location of objects in the room. Using the ground truth mapping results in 5.5% improvement in task success rate. Although the diversity of objects/obstacles in the rooms of ALFRED dataset is high, this improvement is partial. This means our proposed mapping module predicts the maps which is similar to the oracle maps. Also, in Table 4 we conversely compare performance gains provided by our mapping module compared to pure geometric projection for targeted navigation tasks. These results represent quantitative analysis of how much better a graph convolution projected map can obtain compared to pure approximate projection.

**TABLE 4:** Table showing comparison of performance improvement in predicting the class of each node in grid projection map when using Graph convolution filtering over approximate projection map as compared to only approximate projection map.

Map accuracy	Navigable space	Big targets	Medium target	Small targets
Approx. projection	65.1 ± 10	62.3 ± 7	60.2 ± 5	55.3 ± 5
Graph convolution filtering	95.5 ± 2	93.2 ± 2	91.4 ± 3	89.6 ± 5

Values are shown in mean accuracy in percentage of correctly assigned grids followed by standard deviation values. Four major categories are investigated (along columns)- navigable space, identifying grids containing big targets like Bed, identifying medium sized targets like Desk, dresser, and identifying small targets like SideTable, Stool.

Moreover, as mentioned in Section 2.3.1, the intent and slot filling of each sentence is predicted using the language module. To examine the performance of the language module, instead of predicting the instructions intents and target objects (which are the ground truth of our language module that can be extracted from the ALFRED dataset), the ground truth information is replaced in our framework. Even in ground truth data, the target objects of some sentences are not mentioned in the sentence, or some equivalent names are mentioned instead of them. For instance, in the sentence “look left”, the ground truth target object in the ALFRED dataset is considered “Toilet paper hanger”; however, our model predicts nothing for the target object, which actually is correct just by looking at the instruction sentence. Moreover, we prepared a dictionary that links the name mentioned in the sentence to the corresponding ground truth target object when equivalent names are used instead of target objects. Despite the existence of these challenges in predicting the target objects, using the ground truth target objects only improve success rate by 7.1%; proving the promising performance of our Language Module. The accuracy of this model is also reported in Supplementary Table S2 in Supplementary.

Also, the ground truth segmentation mask (pixels) of the target object is replaced with the output of the Cross-Modal Grounding Module. This, in turn, increases mostly the manipulation instructions and causes 9.8% improvement in the task success rate. Disambiguate Module is evaluated by removing this module from the whole framework (since removing this module does not cause missing information and it is basically used to revise the name for target object). As it is shown in Table 3, removing this module results in around 3.0% reduction in task success rate. Thus, among the successful tasks, 3.0% of them contained confused target object names which our Disambiguate module can find their corresponding labels.

## 4 Discussion

A major reason for the significant improvement shown by our modular framework compared to the seq-to-seq models can be attributed to the disentangled manner in which important features of each modality are extracted and combined to obtain high level behaviors. Many end-to-end frameworks, even with sophisticated attention mechanisms, struggle to learn these disentangled representations. This is primarily because of the inherent many-to-one nature of the problem - a language description and a sequence of visual observations can jointly lead to multiple long sequences of action outputs. Therefore, a modular approach that encodes human-level expert semantic knowledge and a generalized understanding of word level semantics can pave the way to train agents with more human like understanding of the tasks and hence, better success rates. Although our technique for finding the slot filling helps our framework to achieve significant performance in finding target objects and navigation commands, we needed to label some instructions for training the language module, which can be considered a limitation. However, we believe our labels would be helpful for other researchers working on ALFRED dataset. As a future work, we will apply popular reinforcement learning techniques for the Planning module by choosing the high-level action given as the input state (outputs of Mapping, Language Understanding, and Cross-Modal Grounding components), and executing a sequence of low level actions.

## Data Availability

Publicly available datasets were analyzed in this study. This data can be found here: https://askforalfred.com/.
