# Supplementary material for: A Modular Vision Language Navigation and Manipulation Framework for Long Horizon Compositional Tasks in Indoor Environment
Source: Front Robot AI. 2022 Jul 13;9:930486. doi: 10.3389/frobt.2022.930486 (PMC9340572; doi:10.3389/frobt.2022.930486)
Supplement: Supplementary file 4 [file DataSheet1.pdf]

# Supplementary Material

## 1 WORKING VIDEOS

Videos demonstrating performance of our proposed framework in the ALFRED dataset is included along with this pdf. Files are named according to room numbers based on baseline authors dataset. Inside each folder, there is video annotated with room number and task number that demonstrates execution of our algorithm. Readers can open the corresponding json files that stores outputs from the language module, namely the input sentence, the predicted intent of the sentence and the predicted slot labels of each word in the sentence. Readers are encouraged to go through the videos and json files in order to get a better understanding of working of our algorithm. Note- the Ai2Thor simulator does not provide panorama vision to the agent, whereas our algorithm needs a much wider field of view which is granted by panorama vision. In order to overcome this difficulty, the agent needs to tilt its camera upwards-downwards and sideways to complete the field of view. To make comparison of trajectory lengths with author baseline fair, we do not count this change of camera tilt as an action that adds towards the trajectory length for both the frameworks (ours and baseline authors).

## 2 MASK AND DEPTH PREDICTION

The depth model takes RGB and depth images from four different directions (front, back, right, and left) of 20 random positions in each room. In total, 8560 RGB images and their corresponding depth images are collected for training and testing. Some results for depth prediction on Alfred dataset are shown in figure S2.

Similar to depth prediction, 8560 data are collected from Ai2thor. Besides, different augmentation techniques such as flipping, rotating, padding, adding noises, changing illumination were used to increase the diversity of data available for training the model. Some sample results on the test dataset for the segmentation task are represented in figure S3, which indicates the effectiveness of this model architecture and our extra preprocessed techniques. Quantitative analysis regarding this module is shown in the ablation studies section.

## 3 GRAPH CONVOLUTION FOR APPROXIMATE PROJECTION MAP REFINEMENT

Our proposed graph convolution framework is applied to approximate projection map to obtain the near perfect representation of the environment around the agent. Each grid node around the agent is classified as either a navigable space, obstacle, target object, or unmappable (beyond wall). Figures S4, S5, S6, S7 presented demonstrate collected input vision data by the agent, approximated projection maps and the corrected map after application of graph convolution algorithm. More information regarding performance of graph convolution framework is presented in perfomrance studies.

## REFERENCES

Anderson, P., Wu, Q., Teney, D., Bruce, J., Johnson, M., Sünderhauf, N., et al. (2018). Vision-and-language navigation: Interpreting visually-grounded navigation instructions in real environments. In *Proceedings of the IEEE Conference on Computer Vision and Pattern Recognition*. 3674–3683

Misra, D., Bennett, A., Blukis, V., Niklasson, E., Shatkhin, M., and Artzi, Y. (2018). Mapping instructions to actions in 3d environments with visual goal prediction. *arXiv preprint arXiv:1809.00786*

Shridhar, M., Thomason, J., Gordon, D., Bisk, Y., Han, W., Mottaghi, R., et al. (2020). Alfred: A benchmark for interpreting grounded instructions for everyday tasks. In *Proceedings of the IEEE/CVF Conference on Computer Vision and Pattern Recognition*. 10740–10749

Wang, X., Huang, Q., Celikyilmaz, A., Gao, J., Shen, D., Wang, Y.-F., et al. (2019). Reinforced cross-modal matching and self-supervised imitation learning for vision-language navigation. In *Proceedings of the IEEE Conference on Computer Vision and Pattern Recognition*. 6629–6638

Wang, X., Xiong, W., Wang, H., and Yang Wang, W. (2018). Look before you leap: Bridging model-free and model-based reinforcement learning for planned-ahead vision-and-language navigation. In *Proceedings of the European Conference on Computer Vision (ECCV)*. 37–53

Zhu, Y., Zhu, F., Zhan, Z., Lin, B., Jiao, J., Chang, X., et al. (2020). Vision-dialog navigation by exploring cross-modal memory. In *Proceedings of the IEEE/CVF Conference on Computer Vision and Pattern Recognition*. 10730–10739

Table S1: Comparison of major techniques in vision language instruction following on previous datasets with the original paper on ALFRED dataset to show the difficulty level of this dataset.

| | Dataset | Dataset complexity | Instruction type | Task type | CNN model | input image | Language model | SR for unseen | year |
|---|---|---|---|---|---|---|---|---|---|
| Seq2seq+PM (Shridhar et al., 2020) | Alfred | 25,743 instructions 428,322 image-action pairs + openable, movable, slicable, cookable, pickable, Togglable objects | Instructions | Navigation Manipulation | ResNet-18 | Each step image | bidirectional LSTM encoder | 0.4 | 2020 |
| CMN (Zhu et al., 2020) | CVDN | 2050 dialogs-7k trajectoies | Question Answering | Navigation | Resnet152 | Panorama | GloVe + 2 layers LSTM | 22.8 | 2020 |
| Mapping with visual goal prediction (Misra et al., 2018) | LANI + CHAI | LANI = 6,000 sequences of natural language instructions CHAI = 1,596 instruction sequences + movable and openable objects | instructions | Navigation + Manipulation | LingUNet (CNN+ leaky ReLU nonlinearities) | Panorama | | LANI = 35.83 CHAI = 40.3 | 2019 |
| RCM + SIL (Wang et al., 2019) | R2R | 21,567 instructions-10,800 panoramic views constructed from 194,400 RGB-D images of 90 building-scale scenes | Instructions | Navigation | ResNet-152 | Panorama | GloVe + finetuning | 60.5 | 2019 |
| RPA (Wang et al., 2018) | R2R | 21,567 instructions-10,800 panoramic views constructed from 194,400 RGB-D images of 90 building-scale scenes | Instructions | Navigation | ResNet152 | Panorama | attention-based LSTM decoder | 24.6 | 2018 |
| R2R data collection paper (Anderson et al., 2018) | R2R | 21,567 instructions-10,800 panoramic views constructed from 194,400 RGB-D images of 90 building-scale scenes | Instructions | Navigation | ResNet-152 | Panorama | LSTM encoder-decoder + reverse order of words | 21.8 | 2018 |

Table S2: Performance for Semantic Segmentation, Depth prediction and NLP models which their ground truth can be extracted from Alfred

| **Module performances** | Metric for evaluation | Value of metric |
|---|---|---|
| Semantic segmentation | IOU | 87.9 |
| Depth prediction | RMS | 0.509 |
| NLP (predicting target objects) | Accuracy | 95.32 |

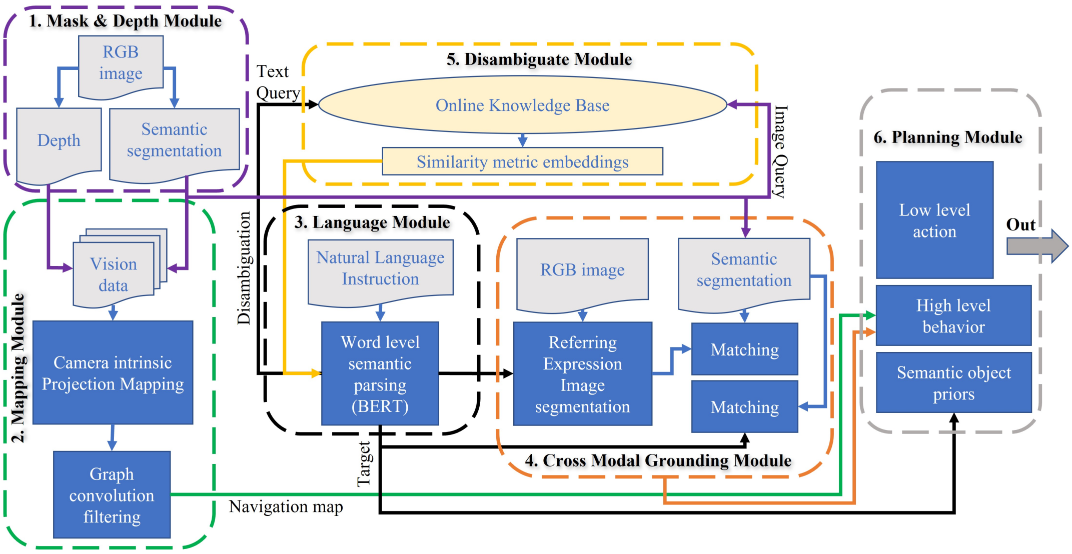


Figure S1: *MoViLan Framework: Different dotted boxes represent various modules and arrows represent transferring information between modules.*

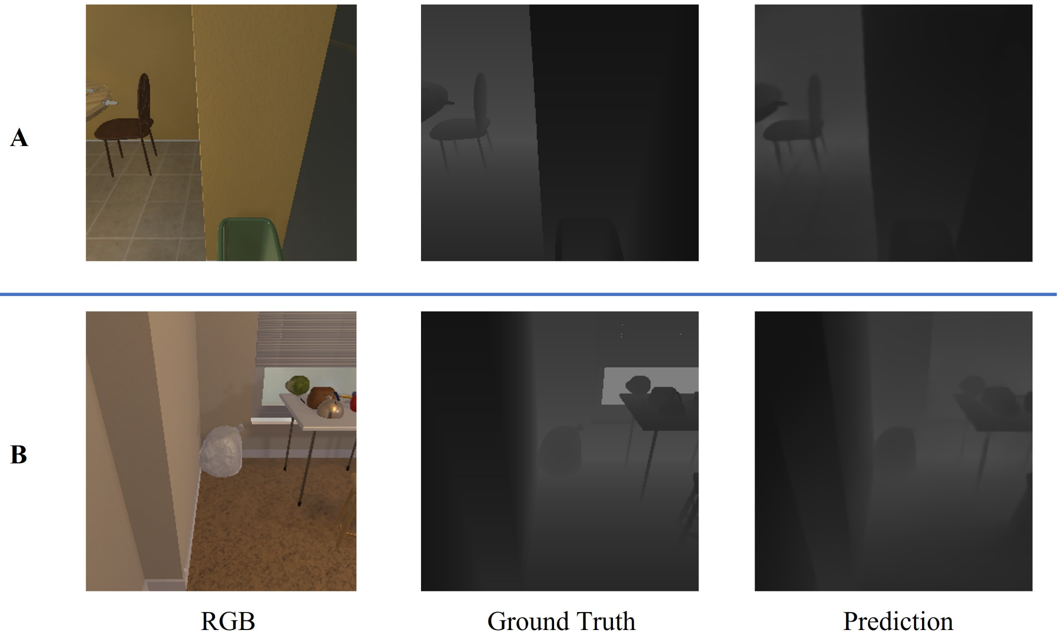


Figure S2: *Depth results: two examples of depth prediction results for a (A) Living room and a (B) Kitchen.*

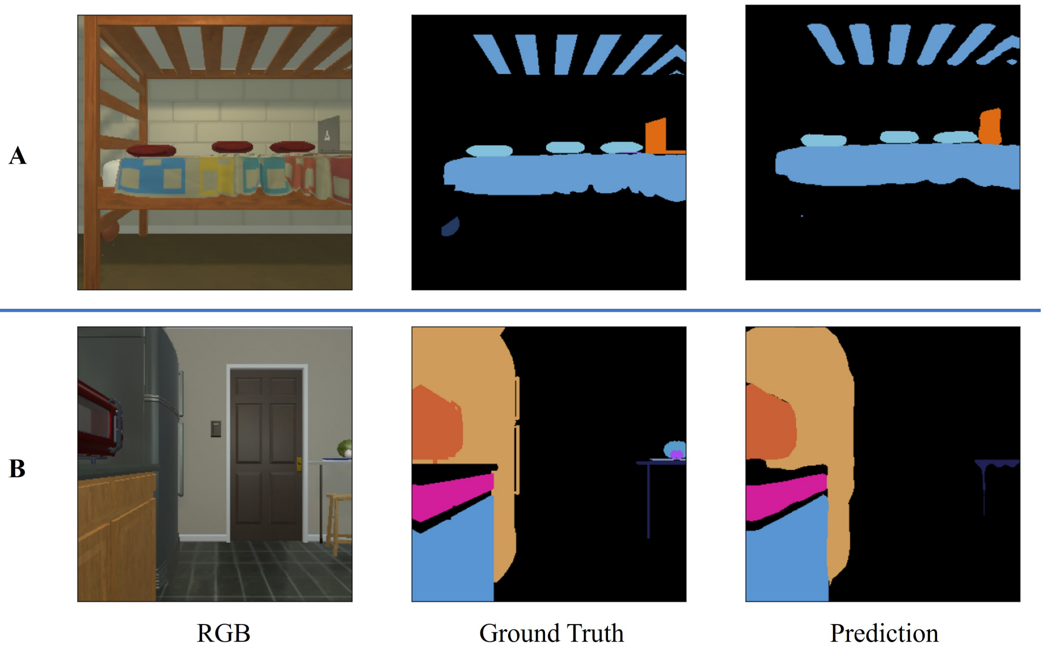


Figure S3: *Segmentation results: two examples of segmentation results for a (A) Bedroom and a (B) Kitchen.*

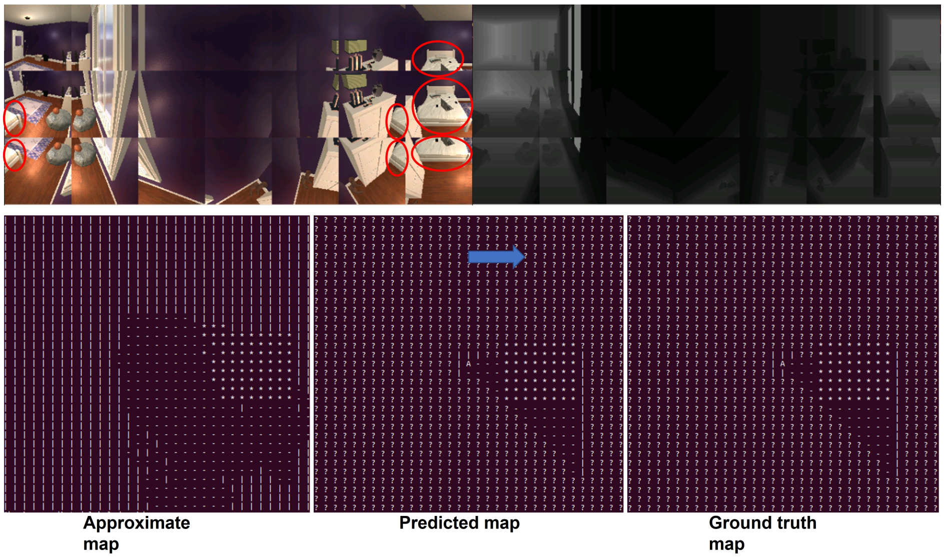


Figure S4: *Captured panorama image by the agent (top left), captured depth normal by the agent (top right), and bottom row showing projected maps. Target object "Bed" is selected in red circle. Bottom row leftmost shows the approximated projection map. Here each grid location in the map is a vector of 4 representing probabilities of that location being a target (marked '*'), navigable space (marked '-'), obstacle (marked 'I') and unknown (because of obstruction, marked '?'). For concise representation, only the argmax value at each grid node is represented by the corresponding marker, meaning if a location has star, it has the highest probability for being the target object. For purposes of planning, unknown is treated the same as obstacle. Input approximate projection (bottom row left) to our proposed Graph convolution filtering algorithm gives the predicted map (bottom row mid). Agent ego centric north is shown by the blue arrow and the ego center (perceived location of self) is shown by the letter 'A'.*

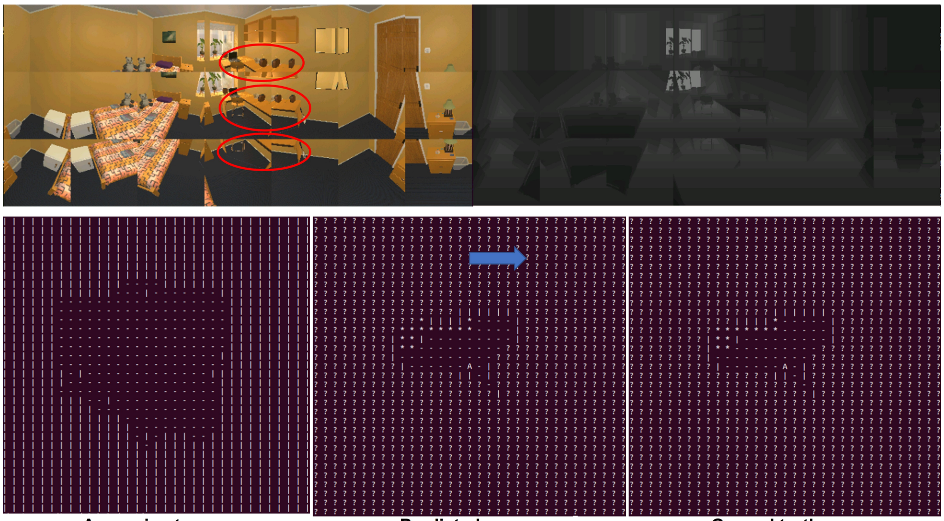


Figure S5: *Captured panorama image by the agent (top left), captured depth normal by the agent (top right), and bottom row showing projected maps. Target object "Desk (L shaped)" is selected in red circle. Bottom row leftmost shows the approximated projection map. Here each grid location in the map is a vector of 4 representing probabilities of that location being a target (marked '*'), navigable space (marked '-'), obstacle (marked 'I') and unknown (because of obstruction, marked '?'). For concise representation, only the argmax value at each grid node is represented by the corresponding marker, meaning if a location has star, it has the highest probability for being the target object. For purposes of planning, unknown is treated the same as obstacle. Input approximate projection (bottom row left) to our proposed Graph convolution filtering algorithm gives the predicted map (bottom row mid). Agent ego centric north is shown by the blue arrow and the ego center (perceived location of self) is shown by the letter 'A'. .*

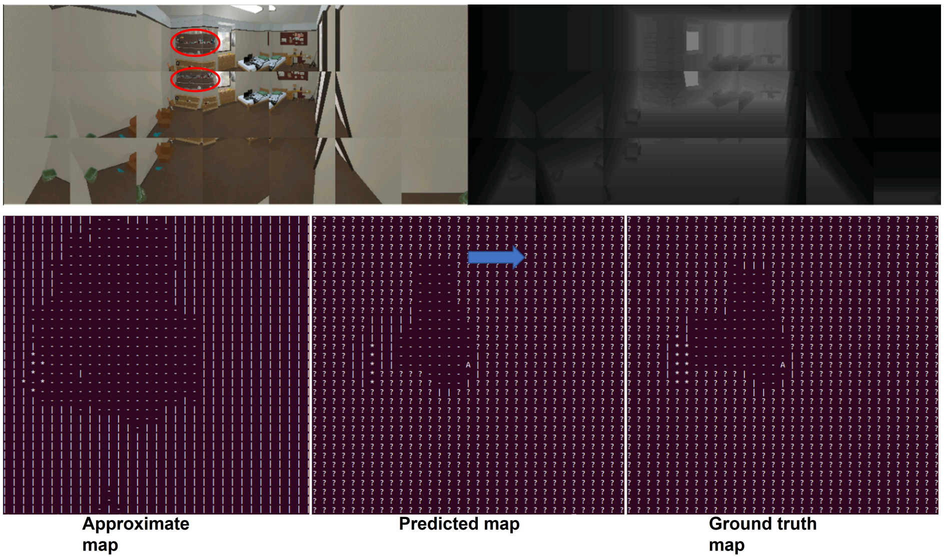


Figure S6: *Captured panorama image by the agent (top left), captured depth normal by the agent (top right), and bottom row showing projected maps. Target object "Shelf" is selected in red circle. Bottom row leftmost shows the approximated projection map. Here each grid location in the map is a vector of 4 representing probabilities of that location being a target (marked '*'), navigable space (marked '-'), obstacle (marked 'I') and unknown (because of obstruction, marked '?'). For concise representation, only the argmax value at each grid node is represented by the corresponding marker, meaning if a location has star, it has the highest probability for being the target object. For purposes of planning, unknown is treated the same as obstacle. Input approximate projection (bottom row left) to our proposed Graph convolution filtering algorithm gives the predicted map (bottom row mid). Agent ego centric north is shown by the blue arrow and the ego center (perceived location of self) is shown by the letter 'A'.*

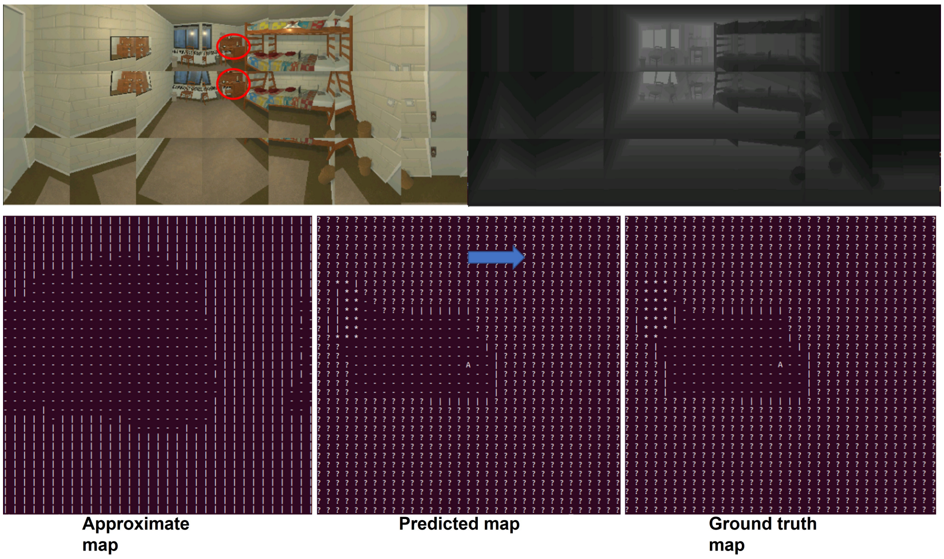


Figure S7: *Captured panorama image by the agent (top left), captured depth normal by the agent (top right), and bottom row showing projected maps. Target object "Dresser" is selected in red circle. Bottom row leftmost shows the approximated projection map. Here each grid location in the map is a vector of 4 representing probabilities of that location being a target (marked '*'), navigable space (marked '-'), obstacle (marked 'I') and unknown (because of obstruction, marked '?'). For concise representation, only the argmax value at each grid node is represented by the corresponding marker, meaning if a location has star, it has the highest probability for being the target object. For purposes of planning, unknown is treated the same as obstacle. Input approximate projection (bottom row left) to our proposed Graph convolution filtering algorithm gives the predicted map (bottom row mid). Agent ego centric north is shown by the blue arrow and the ego center (perceived location of self) is shown by the letter 'A'.*

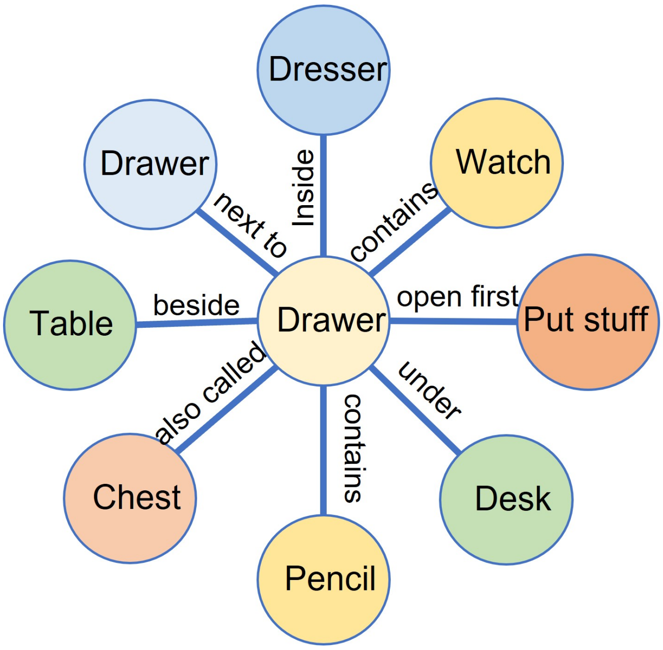


Figure S8: *Illustrative example for knowledge graph for a Drawer object extracted from language corpus of instructions involving drawer.*

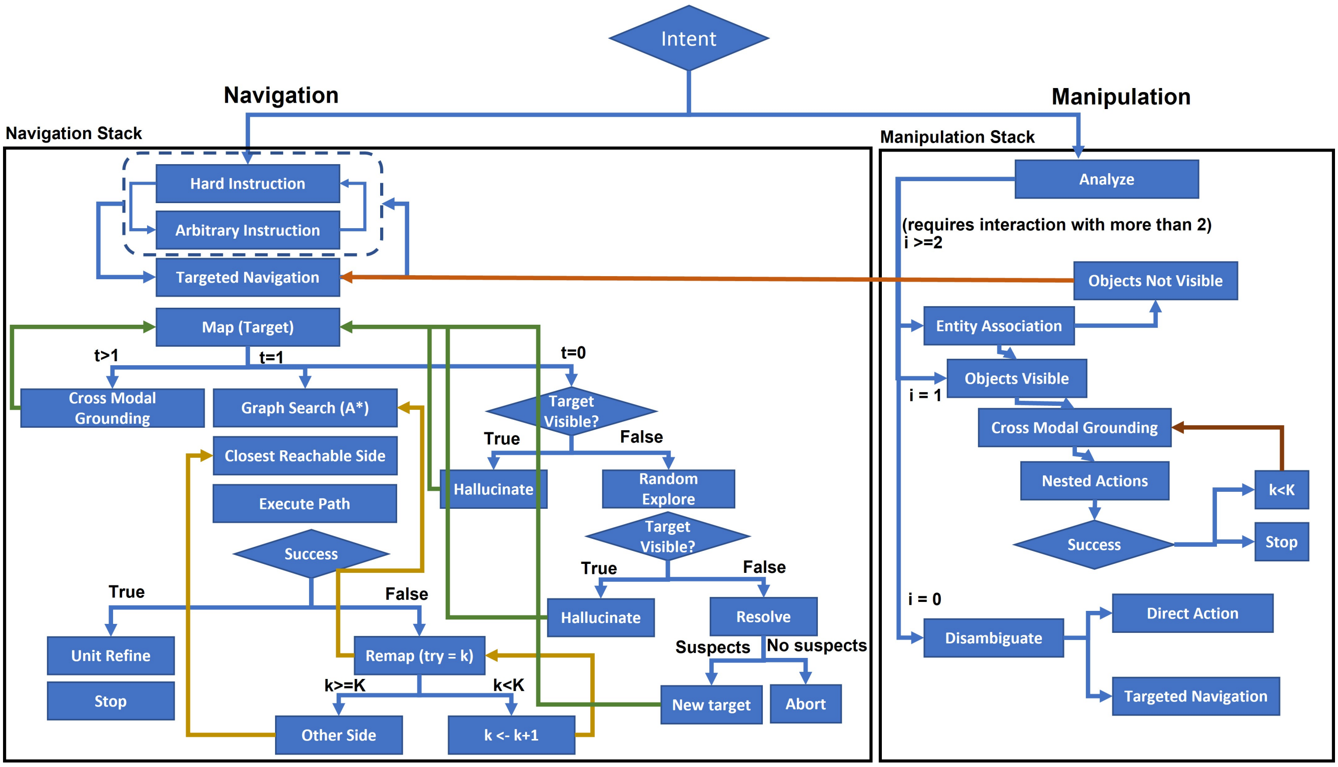


Figure S9: *High level schematic of planning algorithm*
